# Supplementary material for: WNT11-FZD7-DAAM1 signalling supports tumour initiating abilities and melanoma amoeboid invasion
Source: Nat Commun. 2020 Oct 20;11:5315. doi: 10.1038/s41467-020-18951-2 (PMC7575593; doi:10.1038/s41467-020-18951-2)
Supplement: Supplementary file 1 — Supplementary Information [file 41467_2020_18951_MOESM1_ESM.pdf]

SUPPLEMENTARY INFORMATION

WNT11-FZD7-DAAM1 signalling supports tumour initiating abilities and melanoma amoeboid invasion

Irene Rodriguez-Hernandez, Oscar Maiques, Leonie Kohlhammer, Gaia Cantelli, Anna Perdrix, Joanne Monger, Bruce Fanshawe, Victoria L. Bridgeman, Sophia N. Karagiannis, Rosa M. Penin, Joaquim Marcolval, Rosa M. Marti, Xavier Matias-Guiu, Gilbert O. Fruhwirth, Jose L. Orgaz, Ilaria Malanchi and Victoria Sanz-Moreno.

Rodriguez-Hernandez *et al.* Supplementary Figure 1

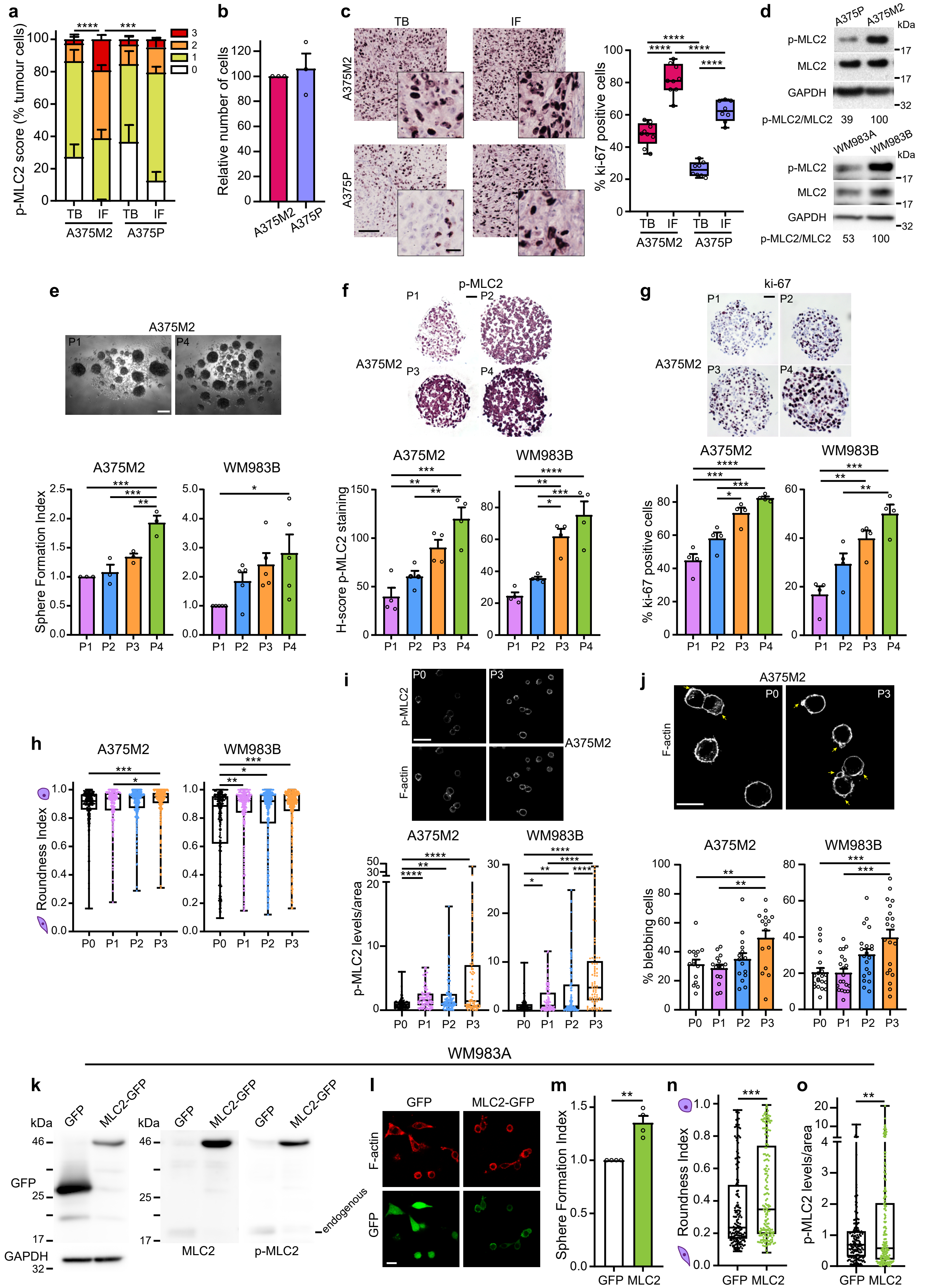

Supplementary Figure 1. Related to Figure 1. Amoeboid cells support tumour initiation in melanoma *in vitro* and *in vivo*

(**a**) Quantification of percentage of cells with score intensity 0-3 for p-MLC2 staining in tumour body (TB) and invasive front (IF) of A375M2 (n=9) and A375P (n=8) tumours from 50,000 cells' condition from Fig.1b. (**b**) Cell viability *in vitro* of A375M2 and A375P cells after 7 days (n=3). (**c**) Representative images (left) and quantification (right) of ki-67 positive cells in TB and IF of A375M2 (n=9) and A375P (n=8) tumours from 50,000 cells' condition from Fig.1b. Scale bar, 100 μm; inset, 25 μm. (**d**) Representative immunoblots of p-MLC2 in the indicated cell lines (n=3). (**e**) Representative phase-contrast images (top) and quantification of sphere formation index (bottom) of A375M2 (n=3) and WM983B cells (n=5) serially passaged. Scale bar, 250 μm. (**f,g**) Representative images (top) and quantification (bottom) of (**f**) H-score of p-MLC2 staining and (**g**) ki-67 positive cells of A375M2 and WM983B spheres serially passaged (n=4). Scale bar, 50 μm. (**h-j**) Representative confocal images (top) and quantification (bottom) of (**h**) cell morphology (>200 cells pooled from n=3), (**i**) p-MLC2 immunofluorescence signal normalized by cell area (>75 cells pooled from n=4) and (**j**) percentage of blebbing cells (5 fields of view per experiment for A375M2 and 10 for WM983B, >85 cells per experiment, n=3) of individual A375M2 and WM983B cells from adherent conditions (P0) and from dissociated cells from spheres serially passaged (P1-P3) on collagen I matrix. Scale bar, (i) 50 μm and (j) 20 μm. Yellow arrows indicate blebs. (**k,l**) (**k**) Representative immunoblots and (**l**) representative confocal images of F-actin (red) and GFP (green) of WM983A cells over-expressing EGFP-wild type MLC2 or control EGFP (n=3). Scale bar, 20 μm. (**m-o**) Quantification of (**m**) sphere formation index (n=4), (**n**) cell morphology (>170 cells pooled from n=3) and (**o**) p-MLC2 immunofluorescence signal normalized by cell area (>165 cells pooled from n=3) of WM983A cells over-expressing EGFP-wild type MLC2 or control EGFP. (a,b,e-g,j,m) Graphs show mean ± s.e.m. (c,h,i,n,o) Box limits show 25th and 75th percentiles, the horizontal line shows the median and whiskers show minimum and maximum range of values. (b,d-o) n means number of independent biological experiments. (a,c,e-g,j) One-way ANOVA with Tukey post-hoc test. (b,m-o) two-tailed t-test. (h,i) Kruskal-Wallis with Dunn's multiple comparison test. For all graphs, *p<0.05, **p<0.01, ***p<0.001, ****p<0.0001. The exact significant p values for *p, **p and ***p are provided in Supplementary Table 1.

Rodriguez-Hernandez *et al.* Supplementary Figure 2

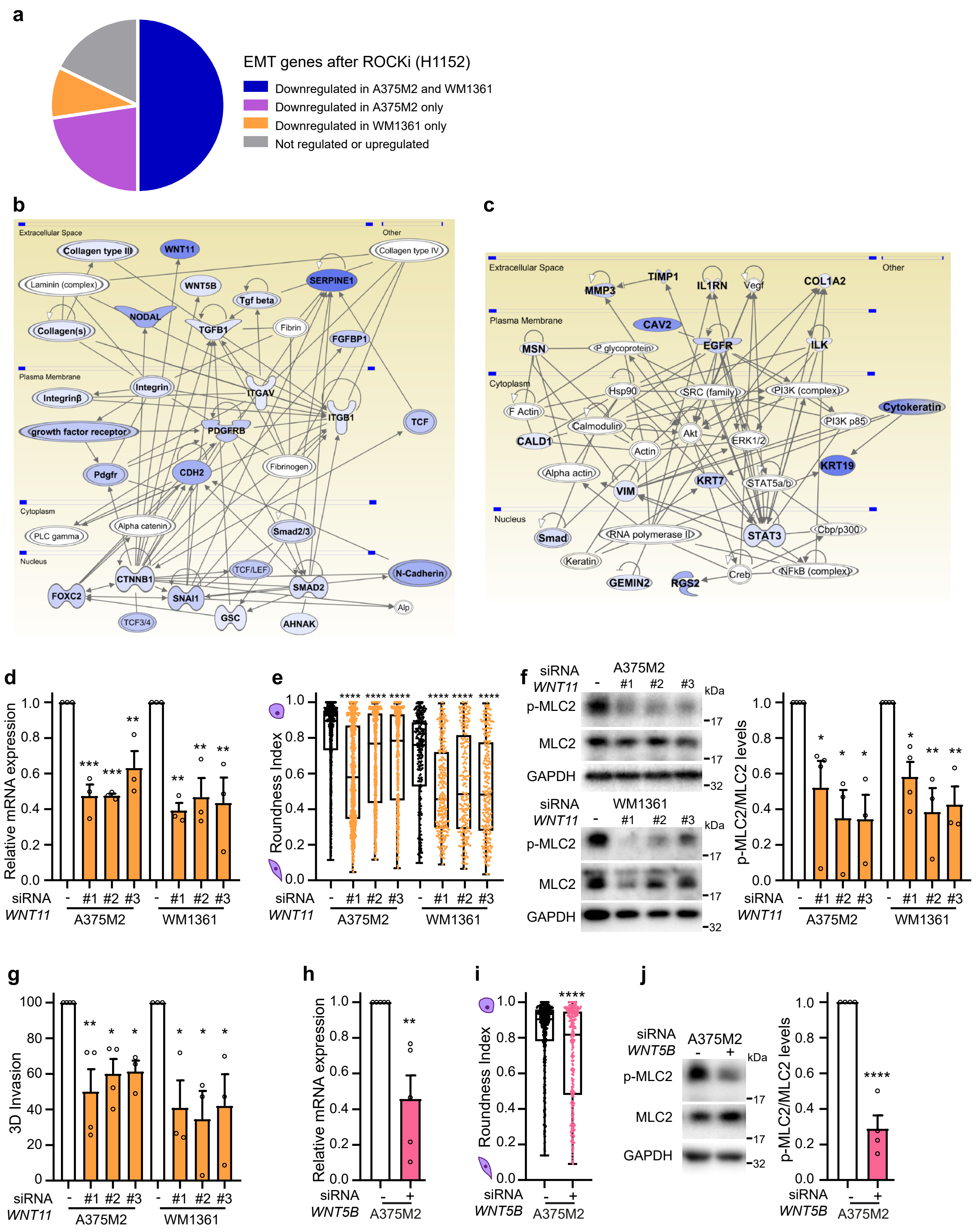

Supplementary Figure 2. Related to Figure 2. EMT genes regulated by ROCK1/2 control amoeboid invasive features

(**a**) Pie chart representing the combined regulation of EMT-related genes in A375M2 and WM1361 cells after 4 h of ROCKi (H1152) (n=4). (**b,c**) Top two enriched networks of downregulated genes in A375M2 and WM1361 cells after 4 h of ROCKi (H1152) predicted by Ingenuity Pathway Analysis software. (**d-g**) After *WNT11* knockdown in A375M2 and WM1361 cells, (**d**) mRNA expression of *WNT11* by qRT-PCR (n=3), (**e**) quantification of cell morphology (>200 cells pooled from n=3), (**f**) representative immunoblots (left) and quantification (right) of p-MLC2 levels (n=4 for *WNT11*#1, n=3 for *WNT11*#2 and *WNT11*#3) and (**g**) 3D invasion index into a collagen I matrix (n=4 for A375M2, n=3 for WM1361). (**h-j**) After *WNT5B* knockdown in A375M2 cells, (**h**) mRNA expression of *WNT5B* by qRT-PCR (n=5), (**i**) quantification of cell morphology (>250 cells pooled from n=3) and (**j**) immunoblots (left) and quantification (right) of p-MLC2 (n=4). (d,f-h,j) Graphs show mean ± s.e.m. (e,i) Box limits show 25th and 75th percentiles, the horizontal line shows the median and whiskers show minimum and maximum range of values. (a,d-j) n means number of independent biological experiments. (d,f,g) One-way ANOVA with Dunnett post-hoc test. (e) Kruskal-Wallis with Dunn's multiple comparison test. (h,j) two-tailed t-test. (i) two-tailed Mann-Whitney test. For all graphs, *p<0.05, **p<0.01, ***p<0.001, ****p<0.0001. The exact significant p values for *p, **p and ***p are provided in Supplementary Table 1.

Rodriguez-Hernandez *et al.* Supplementary Figure 3

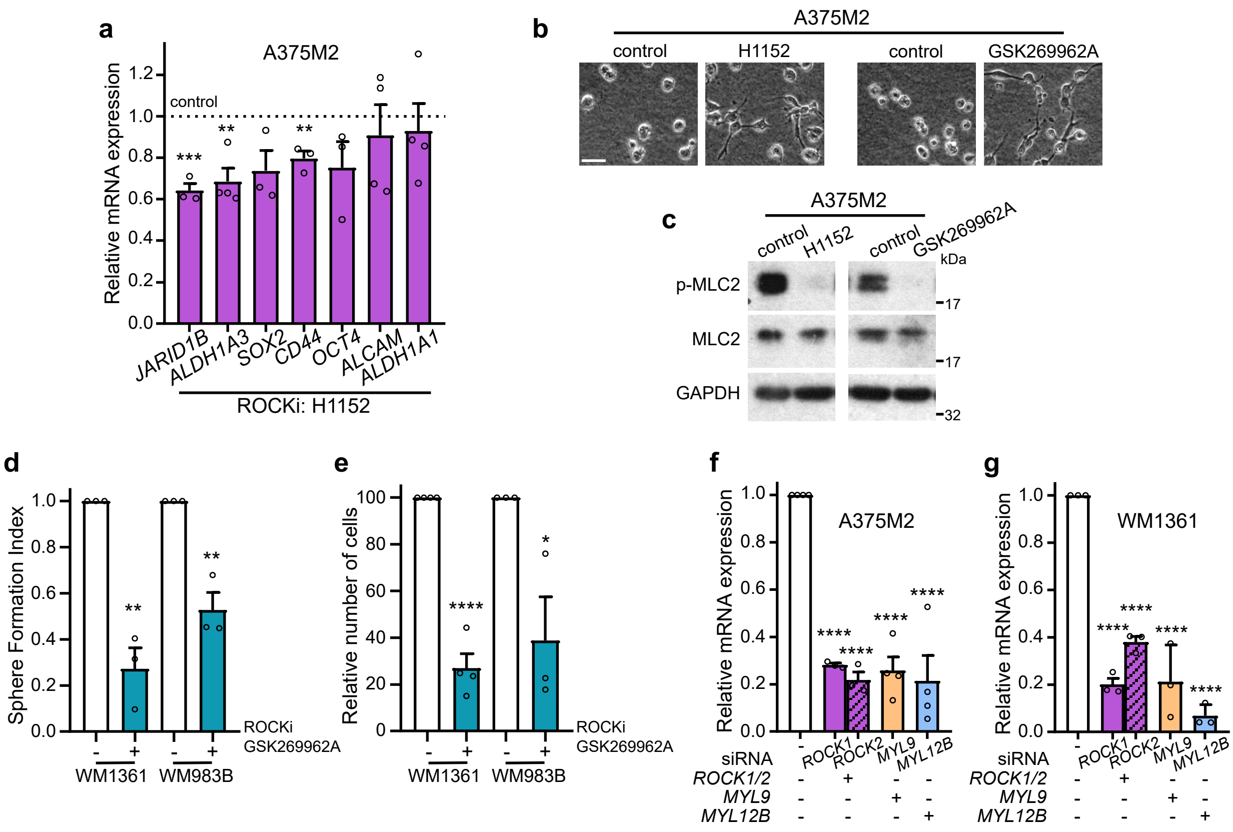

Supplementary Figure 3. Related to Figure 3. Non-canonical Wnt ligands support melanosphere formation and amoeboid behaviour

(**a**) mRNA expression of stem cell-related markers by qRT-PCR in A375M2 cells treated with ROCKi (H1152) for 24 h compared to control A375M2 cells (n=3 for *JARID1B*, *SOX2, OCT4*, *CD44;* n=4 for *ALDH1A3*, *ALCAM*, *ALDH1A1*). (**b**) Representative phase-contrast images and (**c**) immunoblots of p-MLC2 in A375M2 cells after 24 h treatment with ROCKi (H1152 or GSK269962A) (n=3). Scale bar, 50µm. (**d-e**) Quantification of (**d**) sphere formation index (n=3) and (**e**) cell viability (n=4 for WM1361, n=3 for WM983B) of WM1361 and WM983B cells treated with one dose of ROCKi (GSK269962A). (**f,g**) mRNA expression of indicated genes by qRT-PCR in (**f**) A375M2 (n=4) and (**g**) WM1361 cells (n=3) after *ROCK1/2*, *MYL9* or *MYL12B* knockdown. (a,d-g) Graphs show mean ± s.e.m. (a-g) n means number of independent biological experiments. (a,d,e) two-tailed t-test. (f,g) One-way ANOVA with Dunnett post-hoc test. For all graphs, **p*<0.05, ***p*<0.01, ****p*<0.001, *****p*<0.0001. The exact significant p values for **p*, ***p* and ****p* are provided in Supplementary Table 1.

Rodriguez-Hernandez *et al*. Supplementary Figure 4

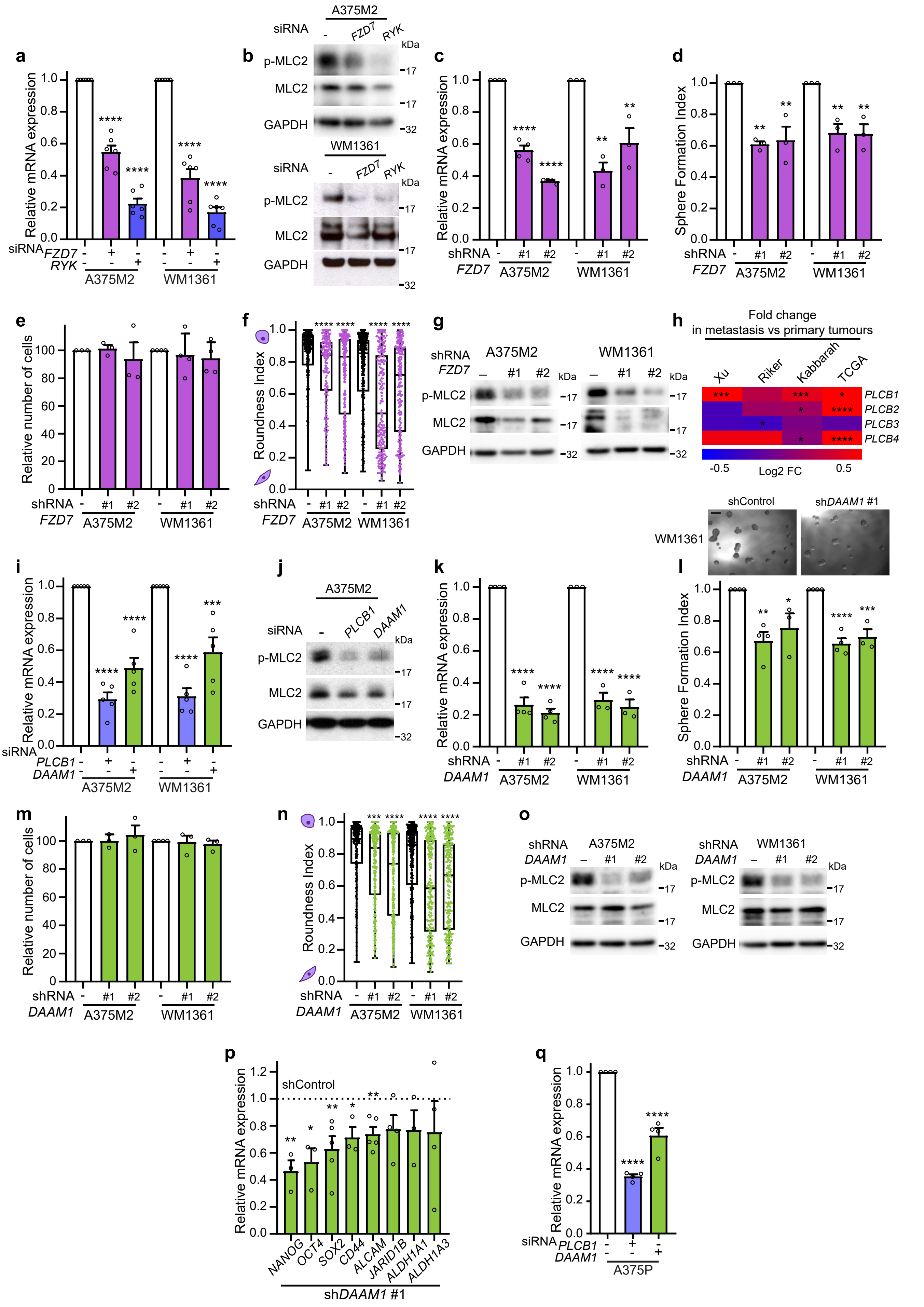

Supplementary Figure 4. Related to Figure 4. FZD7 downstream of WNT11 supports melanosphere formation and amoeboid invasion *via* DAAM1

(**a,b**) After *FZD7* or *RYK* knockdown in A375M2 and WM1361 cells, (**a**) mRNA expression of indicated genes by qRT-PCR (n=6) and (**b**) representative immunoblots of p-MLC2 (n=3 for A375M2, n=4 for WM1361). (**c-g**) (**c**) mRNA expression of *FZD7* by qRT-PCR (n=4 for A375M2, n=3 for WM1361), quantification of (**d**) sphere formation index (n=3), (**e**) cell viability (n=3 for A375M2, n=4 for WM1361) and (**f**) cell morphology (>200 cells pooled from n=3) and (**g**) representative immunoblots of p-MLC2 (n=3) in A375M2 and WM1361 cells expressing non-silencing shRNA (shControl) and two different shRNAs against *FZD7* (sh*FZD7*). (**h**) Heatmap representing log2 fold change in *PLCB* isoforms' expression in metastatic *versus* primary melanoma samples. Raw data were obtained from GEO and TCGA databases. (**i,j**) After *PLCB1* or *DAAM1* knockdown in A375M2 and WM1361 cells, (**i**) mRNA expression of indicated genes by qRT-PCR (n=5) and (**j**) representative immunoblots of p-MLC2 (n=3). (**k-p**) (**k**) mRNA expression of *DAAM1* by qRT-PCR (n=5), (**l**) representative phase-contrast images (scale bar, 250 μm) (top) and quantification of sphere formation index (bottom) (n=4 for sh*DAAM1*#1, n=3 for sh*DAAM1*#2), (**m**) cell viability (n=3 for A375M2, n=4 for WM1361), (**n**) cell morphology (>200 cells pooled from n=3), (**o**) representative immunoblots of p-MLC2 (n=3) and (**p**) mRNA expression of stem cell-related markers by qRT-PCR (n=3 for *NANOG*, *OCT4*, *CD44*, *ALDHA1A*; n=4 for *JARID1B*, *ALDH1A3*; n=5 for *SOX2*, *ALCAM*) in A375M2 and WM1361 cells expressing shControl and two different shRNA against *DAAM1* (sh*DAAM1*). (**q**) mRNA expression of indicated genes by qRT-PCR in A375P cells after *PLCB1* or *DAAM1* knockdown (n=4). (a,c-e,i,k-m,p,q) Graphs show mean ± s.e.m. (f,n) Box limits show 25th and 75th percentiles, the horizontal line shows the median and whiskers show minimum and maximum range of values. (a-g,i-q) n means number of independent biological experiments. (a,c-e,i,k-m,q) One-way ANOVA with Dunnett post-hoc test. (f,n) Kruskal-Wallis with Dunn's multiple comparison test. (h) two-tailed t-test with Welch's correction. (p) two-tailed t-test. For all graphs, *p<0.05, **p<0.01, ***p<0.001, ****p<0.0001. The exact significant p values for *p, **p and ***p are provided in Supplementary Table 1.

Rodriguez-Hernandez *et al.* Supplementary Figure 5

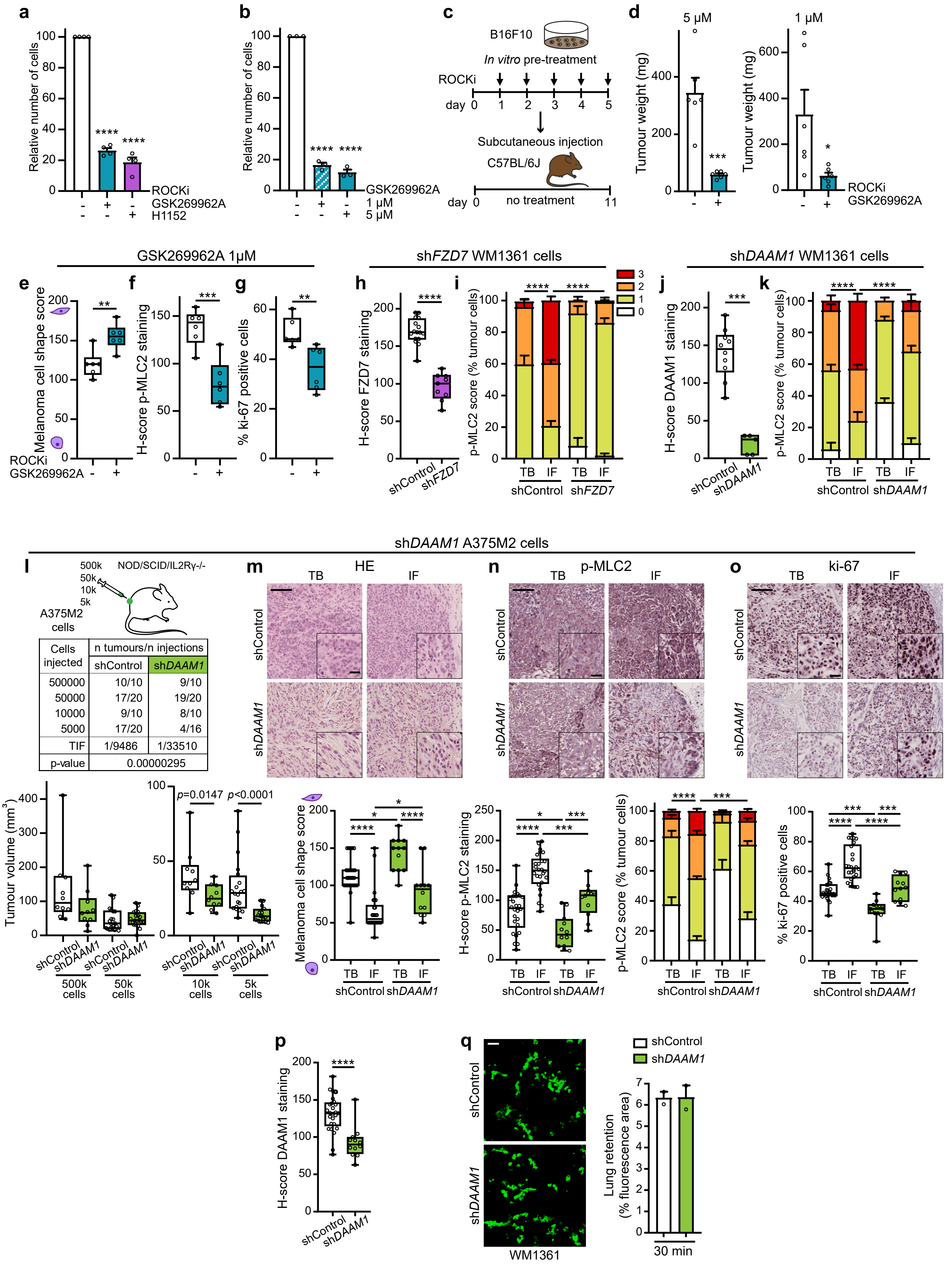

Cells injected	n tumours/n injections	
	shControl	sh*DAAM1*
500000	10/10	9/10
50000	17/20	19/20
10000	9/10	8/10
5000	17/20	4/16
TIF	1/9486	1/33510
p-value	0.00000295	

Supplementary Figure 5. Related to Figure 5. FZD7-DAAM1-RhoA-ROCK1/2 supports tumour initiation and metastasis *in vivo*

(**a**) Cell viability of A375M2 cells treated with ROCKi (5µM H1152 or 5µM GSK269962A) for 5 days *in vitro* (n=4). (**b**) Cell viability of B16F10 cells treated with ROCKi (5µM or 1µM GSK269962A) for 5 days *in vitro* (n=3). (**c,d**) (**c**) Schematic of experiment and (**d**) tumour weight of ROCKi (5µM or 1µM GSK269962A) pre-treated B16F10 cells 11 days post-subcutaneous injection in C57BL/6J mice (n=6 mice/group). (**e-g**) Quantification of (**e**) melanoma cell shape score, (**f**) H-score of p-MLC2 staining and (**g**) ki-67 positive cells in B16F10 tumours (n=6 tumours/group) from (c). (**h,i**) Quantification of (**h**) FZD7 staining and (**i**) percentage of cells with score intensity 0-3 for p-MLC2 staining in shControl (n=16) and sh*FZD7* (n=9) WM1361 derived tumours from 50,000 cells' condition from Fig.5e. (**j,k**) Quantification of (**j**) DAAM1 staining and (**k**) percentage of cells with score intensity 0-3 for p-MLC2 staining in shControl (n=10) and sh*DAAM1* (n=5) WM1361 derived tumours from 50,000 cells' condition from Fig.5i. (**l**) Limiting dilution assay estimating tumour initiating frequency (TIF) (top) and tumour volume (bottom) of shControl and sh*DAAM1* A375M2 cells when injected at different dilutions (500,000, 50,000, 10,000 and 5,000 cells) in NOD/SCID/IL2Rγ-/- (NSG) mice (Number of tumours per condition indicated in table). TIF was determined using ELDA. (**m-o**) Representative images (top) and quantification (bottom) of (**m**) melanoma cell shape score, (**n**) H-score and percentage of cells with score intensity 0-3 for p-MLC2 staining and (**o**) ki-67 positive cells in TB and IF of shControl (n=26) and sh*DAAM1* (n=12) derived tumours from 10,000 and 5,000 cells' conditions from (l). Scale bar, 100 µm; inset, 25 µm. (**p**) Quantification of DAAM1 staining in shControl (n=26) and sh*DAAM1* (n=12) A375M2 derived tumours from 10,000 and 5,000 cells' conditions from (l). (**q**) Representative confocal images (left) and percentage of fluorescence area (right) of mouse lungs 30 min after tail vein injection of WM1361 cells expressing shControl or sh*DAAM1* constructs (n=2 mice/group). Scale bar, 50µm. (a,b,d,i,k,q) Graphs show mean ± s.e.m. (e-h,j,l-p) Box limits show 25th and 75th percentiles, the horizontal line shows the median and whiskers show minimum and maximum range of values. (a,b) One-way ANOVA with Dunnett post-hoc test. (d-h,q) two-tailed t-test. (j,l,p) two-tailed Mann-Whitney test. (i,k,m-o) One-way ANOVA with Tukey post-hoc test. For all graphs, *p<0.05, **p<0.01, ***p<0.001, ****p<0.0001. The exact significant p values for *p, **p and ***p are provided in Supplementary Table 1. Schematics in this figure were created using Servier Medical Art templates licensed under a Creative Commons Attribution 3.0 Unported License (https://smart.servier.com).

Rodriguez-Hernandez *et al*. Supplementary Figure 6

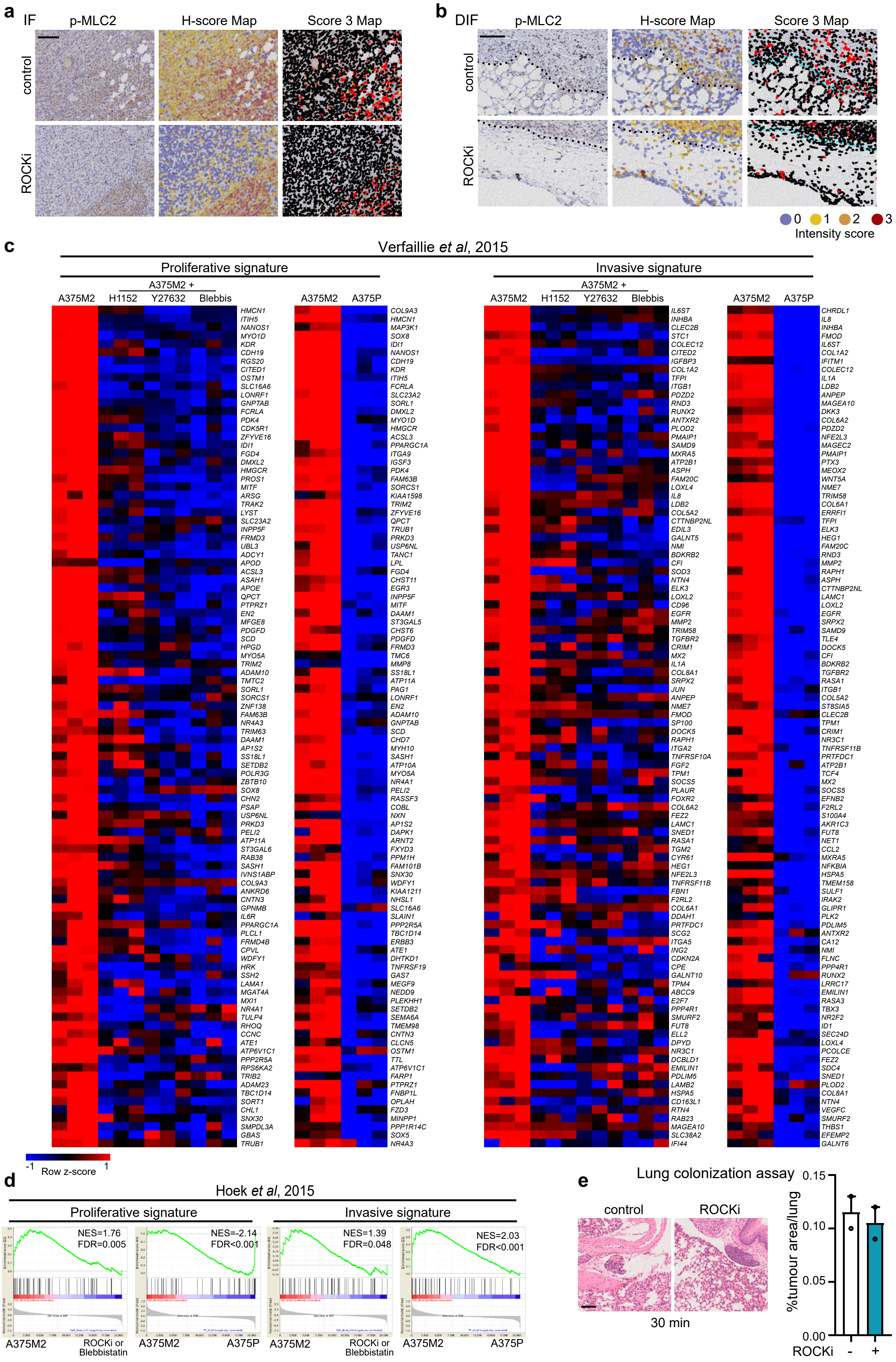

Supplementary Figure 6. Related to Figure 6. Amoeboid behaviour enhances tumour formation, tumour progression and metastasis *in vivo*

(**a,b**) Representative images of score intensity 0-3 for p-MLC2 staining in (**a**) IF and (**b**) distal invasive front (DIF) from primary tumours from Fig.6a. Dashed lines represent the boundary between IF and DIF. Scale bar, 50 µm. (**c**) Heatmaps showing expression of top 100 proliferative and invasive genes from Verfaille study[1] enriched in A375M2 cells compared to A375M2 cells treated with ROCKi (H1152, Y27632) and blebbistatin or to A375P cells[2]. (**d**) GSEA plots showing enrichment of proliferative and invasive gene signatures from Hoek study[3] in A375M2 cells compared to A375M2 cells treated with ROCKi (H1152, Y27632) and blebbistatin or to A375P cells[2]. NES, normalized enrichment score; FDR, false discovery rate. (**e**) Representative images of mouse lungs (left) and quantification of tumour area (right) 30 min after tail vein injection of 4599 cells pre-treated with ROCKi (5µM GSK269962A) for 24 h in NSG mice (n=2 mice/group). Scale bar, 100 µm. (e) Graphs show mean ± s.e.m. (e) two-tailed Mann-Whitney test.

Rodriguez-Hernandez *et al*. Supplementary Figure 7

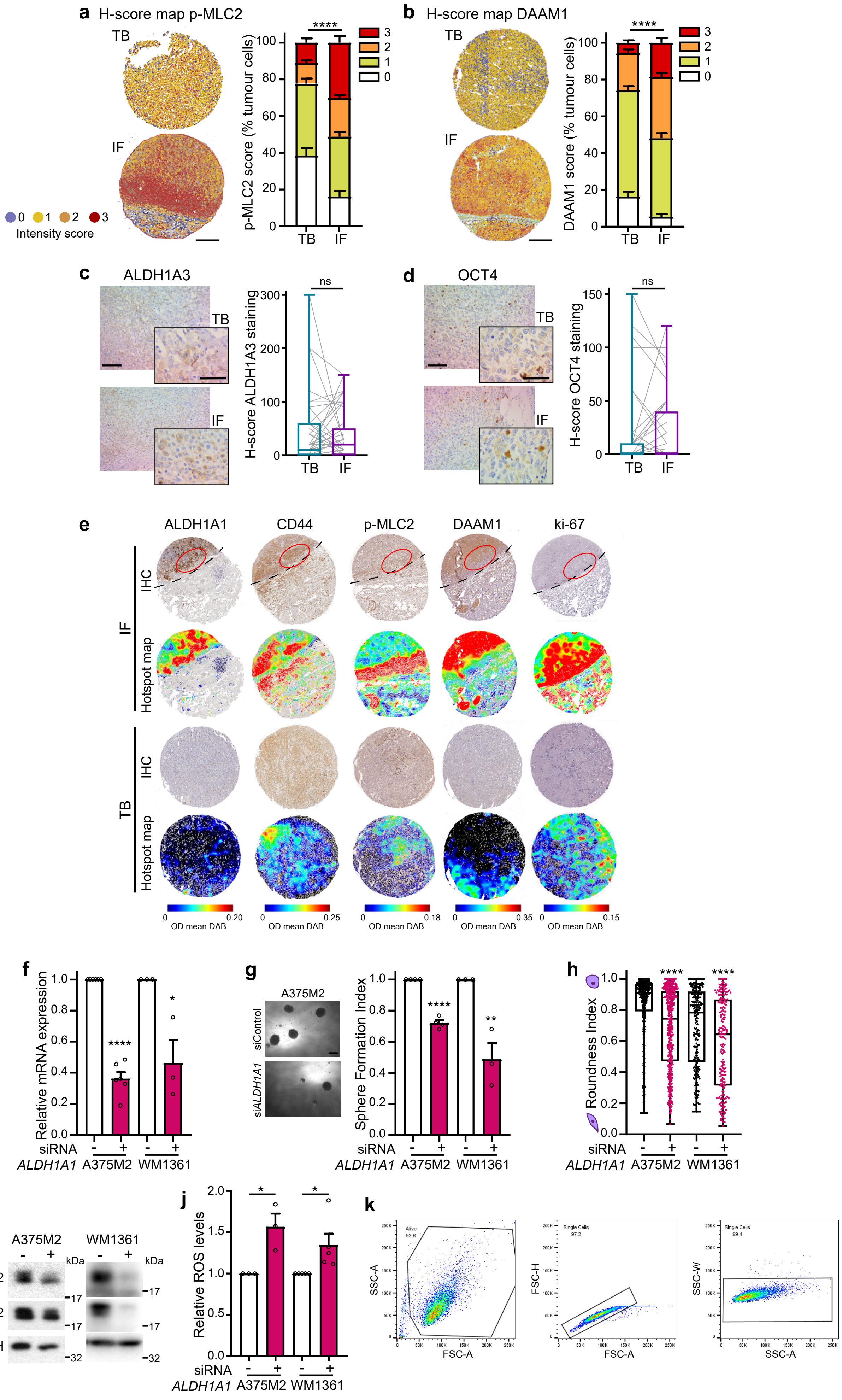

Supplementary Figure 7. Related to Figure 7. Analysis of the invasive front of human primary melanomas

(**a,b**) Representative images (left) and quantification (right) of percentage of cells with score intensity 0-3 for (**a**) p-MLC2 staining and (**b**) DAAM1 staining in matched TB and IF from primary melanomas. Scale bar, 300 µm. (**c,d**) Representative images (left) and quantification (right) of (**c**) ALDH1A3 and (**d**) OCT4 in matched TB and IF from primary melanomas. Scale bar, 100 µm; inset, 50 µm. (a-d) 53 primary melanomas. (**e**) IHC images and hotspot maps for ALDH1A1, CD44, p-MLC2, DAAM1 and ki-67 stainings in consecutive sections of IF and TB areas from a human primary melanoma. Red circles show the areas with higher spatial correlation. (**f-j**) After *ALDH1A1* knockdown in A375M2 and WM1361 cells, (**f**) mRNA expression of *ALDH1A1* by qRT-PCR (n=6 for A375M2, n=3 for WM1361), (**g**) representative phase-contrast images (scale bar, 250 µm) (left) and quantification of sphere formation index (right) (n=4 for A375M2, n=3 for WM1361), (**h**) cell morphology (>200 cells pooled from n=3), (**i**) representative immunoblots of p-MLC2 (n=3) and (**j**) reactive oxygen species (ROS) measurement (n=3 for A375M2, n=5 for WM1361). (**k**) Gating strategy for intracellular measurement of ROS. (a,b,f,g,j) Graphs show mean ± s.e.m. (c,d,h) Box limits show 25th and 75th percentiles, the horizontal line shows the median and whiskers show minimum and maximum range of values. (f-j) n means number of independent biological experiments. (a-d) two-tailed Wilcoxon test. (f,g,j) two-tailed t-test. (h) two-tailed Mann-Whitney test. For all graphs, *p<0.05, **p<0.01, ***p<0.001, ****p<0.0001. The exact significant p values for *p, **p and ***p are provided in Supplementary Table 1.

Rodriguez-Hernandez *et al.* Supplementary Figure 8

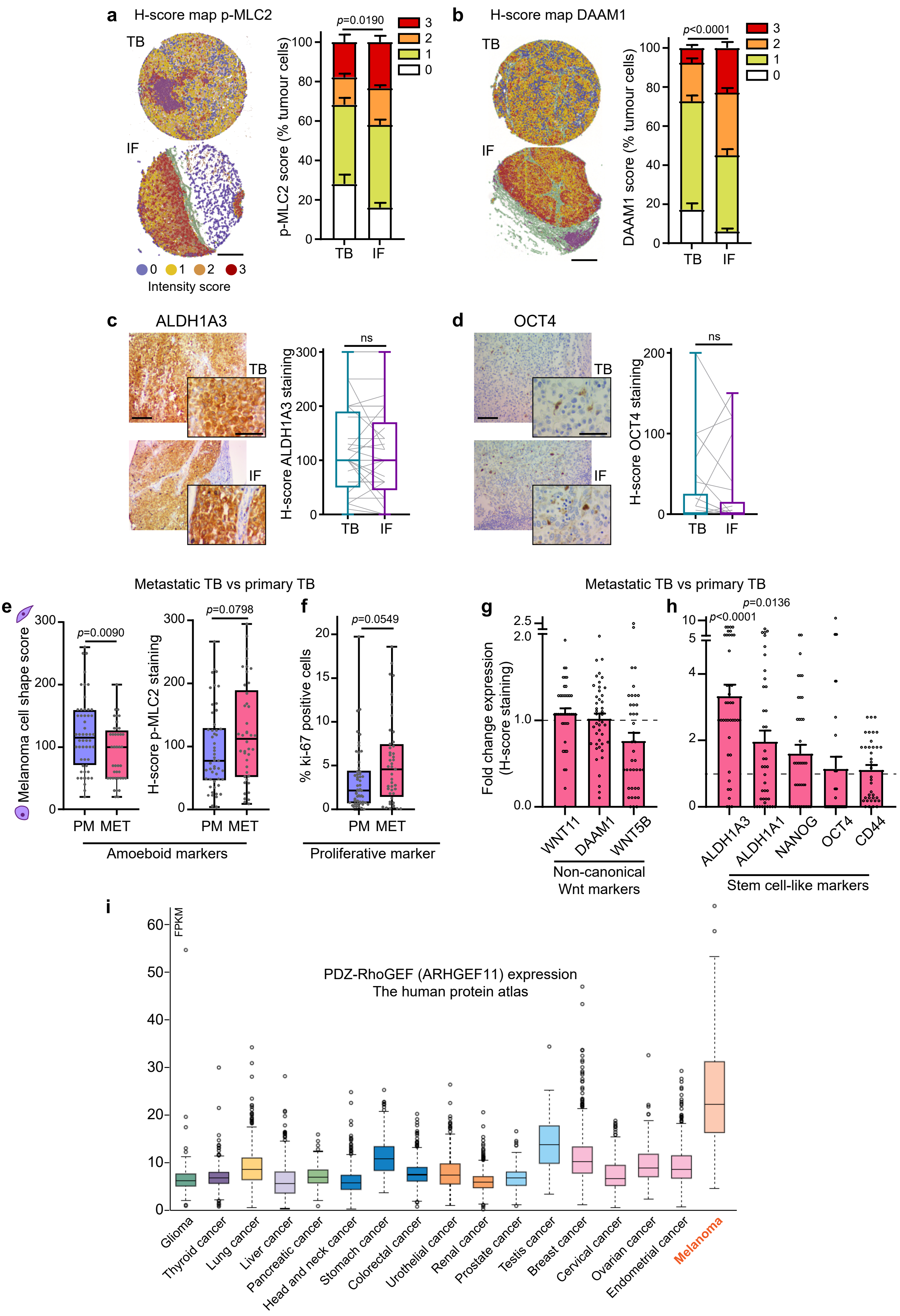

Supplementary Figure 8. Related to Figure 8. Analysis of the invasive front of human metastatic melanomas

(**a,b**) Representative images (left) and quantification (right) of percentage of cells with score intensity 0-3 for (**a**) p-MLC2 staining and (**b**) DAAM1 staining in matched TB and IF from melanoma metastasis. Scale bar, 300 µm. (**c,d**) Representative images (left) and quantification (right) of (**c**) ALDH1A3 and (**d**) OCT4 in matched TB and IF from melanoma metastasis. Scale bar, 100 µm; inset, 50 µm. (a-d) 45 metastatic melanomas. (**e-h**) Fold change expression of (**e**) amoeboid markers, (**f**) ki-67 proliferative marker, (**g**) non-canonical Wnt markers and (**h**) stem cell-like markers in TB of melanoma metastasis compared to TB of primary tumours. (e-h) 53 primary and 45 metastatic melanomas. (**i**) RNA-seq expression data of PDZ-RhoGEF (ARHGEF11) across tumour types from the human protein atlas (www.proteinatlas.org). (a,b,g,h) Graphs show mean ± s.e.m. (c-f) Box limits show 25th and 75th percentiles, the horizontal line shows the median and whiskers show minimum and maximum range of values. (i) Box limits show 25th and 75th percentiles, the horizontal line shows the median and points are displayed as outliers if they are above or below 1.5 times the interquartile range. (a-d) two-tailed Wilcoxon test. (e,g) two-tailed t-test. (f,h) two-tailed Mann-Whitney test.

Supplementary Table 1. Exact significant p values from figures

Figure 1c

Comparison	Exact p value
500k A375M2 vs. A375P	*p=0.0255
50k A375M2 vs. A375P	*p=0.0105
5k A375M2 vs. A375P	**p=0.0071

Figure 1d

Comparison	Exact p value
A375M2 TB vs. A375M2 IF	****p<0.0001
A375M2 TB vs. A375P TB	**p=0.0049
A375M2 IF vs. A375P IF	****p<0.0001
A375P TB vs. A375P IF	***p=0.0004

Figure 1e

Comparison	Exact p value
A375M2 TB vs. A375M2 IF	****p<0.0001
A375M2 IF vs. A375P IF	**p=0.0016
A375P TB vs. A375P IF	*p=0.0265

Figure 1f

Comparison	Exact p value
A375P P1 vs. P3	*p=0.0185
A375P P1 vs. P4	**p=0.004
A375P P2 vs. P4	*p=0.0493
WM983A P1 vs. P3	*p=0.0168
WM983A P1 vs. P4	***p=0.0008
WM983A P2 vs. P4	**p=0.0025

Figure 1g

Comparison	Exact p value
A375P P1 vs. P3	*p=0.0393
A375P P1 vs. P4	**p=0.0023
A375P P2 vs. P4	*p=0.0141
WM983A P1 vs. P3	***p=0.0001
WM983A P1 vs. P4	****p<0.0001
WM983A P2 vs. P3	*p=0.0126
WM983A P2 vs. P4	****p<0.0001
WM983A P3 vs. P4	*p=0.0312

Figure 1h

Comparison	Exact p value
A375P P1 vs. P3	***p=0.0004
A375P P1 vs. P4	***p=0.0001
A375P P2 vs. P3	**p=0.0079
A375P P2 vs. P4	**p=0.0018
WM983A P1 vs. P3	**p=0.0059
WM983A P1 vs. P4	***p=0.0003
WM983A P2 vs. P4	**p=0.0042

Figure 1i

Comparison	Exact p value
A375P P0 vs. P1	****p<0.0001
A375P P0 vs. P2	****p<0.0001
A375P P0 vs. P3	****p<0.0001
A375P P1 vs. P2	****p<0.0001
A375P P1 vs. P3	*p=0.0348
WM983A P0 vs. P1	****p<0.0001
WM983A P0 vs. P2	****p<0.0001
WM983A P0 vs. P3	****p<0.0001
WM983A P1 vs. P2	****p<0.0001
WM983A P1 vs. P3	****p<0.0001

Figure 1j

Comparison	Exact p value
A375P P0 vs. P2	****p<0.0001
A375P P0 vs. P3	**p=0.001
A375P P1 vs. P2	****p<0.0001
A375P P1 vs. P3	****p<0.0001
WM983A P0 vs. P1	****p<0.0001
WM983A P0 vs. P2	****p<0.0001
WM983A P0 vs. P3	****p<0.0001
WM983A P1 vs. P2	**p=0.0054
WM983A P1 vs. P3	****p<0.0001

Figure 1k

Comparison	Exact p value
A375P P0 vs. P3	***p=0.0007
A375P P1 vs. P3	*p=0.0282
WM983A P0 vs. P2	**p=0.0039
WM983A P0 vs. P3	**p=0.0031
WM983A P1 vs. P2	**p=0.0044
WM983A P1 vs. P3	**p=0.0035

Figure 2c

**Comparison A375M2**	**Exact p value**
KRT19	****p<0.0001
WNT11	****p<0.0001
RGS2	****p<0.0001
GSC	****p<0.0001
CAV2	****p<0.0001
SERPINE1	****p<0.0001
SNAI1	****p<0.0001
CAMK2N1	****p<0.0001
TCF3	****p<0.0001
JAG1	****p<0.0001
NODAL	****p<0.0001
FOXC2	****p<0.0001
STEAP1	***p=0.0001
CTNNB1	***p=0.0001
ITGA5	***p=0.0001
MMP3	***p=0.0002
PLEK2	***p=0.0002
AHNAK	***p=0.0002
PDGFRB	***p=0.0003
VIM	***p=0.0003
STAT3	***p=0.0004
SOX10	***p=0.0005
WNT5B	***p=0.0005
TGFB3	***p=0.0005
TGFB1	***p=0.0005
IGFBP4	***p=0.0006
TSPAN13	***p=0.0007
MMP2	***p=0.0009
FGFBP1	**p=0.0011
SNAI3	**p=0.0012
CDH2	**p=0.0013
KRT7	**p=0.0019
IL1RN	**p=0.0035
TIMP1	**p=0.004
COL1A2	**p=0.0053
ZEB1	**p=0.006
CDH1	**p=0.0064
NUDT13	**p=0.007
SMAD2	**p=0.007
NOTCH1	**p=0.0072
TCF4	**p=0.0077
EGFR	**p=0.0082
GNG11	*p=0.0101
VCAN	*p=0.0102
ITGB1	*p=0.0105
COL5A2	*p=0.0114
ILK	*p=0.0115
AKT1	*p=0.0117
TFPI2	*p=0.0126
CALD1	*p=0.013
DESI1	*p=0.0131
GEMIN2	*p=0.0142
OCLN	*p=0.0159
RAC1	*p=0.018
MSN	*p=0.0189
F11R	*p=0.0208
ITGAV	*p=0.048

**Comparison WM1361**	**Exact p value**
SERPINE1	****p<0.0001
IGFBP4	****p<0.0001
WNT11	****p<0.0001
CDH2	***p=0.0002
CAV2	***p=0.0003
TCF4	***p=0.0004
F11R	***p=0.0008
FGFBP1	**p=0.0011
GEMIN2	**p=0.0013
TIMP1	**p=0.0017
FN1	**p=0.002
DESI1	**p=0.0021
RGS2	**p=0.0023
KRT7	**p=0.0028
PDGFRB	**p=0.0031
EGFR	**p=0.0047
DSP	**p=0.0049
TGFB2	**p=0.007
COL1A2	**p=0.0098
CALD1	*p=0.0106
MSN	*p=0.0111
MMP3	*p=0.0128
ILK	*p=0.0136
ITGB1	*p=0.0177
TFPI2	*p=0.0181
SMAD2	*p=0.0181
SNAI2	*p=0.0195
TCF3	*p=0.0222
IL1RN	*p=0.0227
TGFB1	*p=0.0238
NODAL	*p=0.0239
TMEFF1	*p=0.0239
KRT14	*p=0.024
MMP2	*p=0.0258
NUDT13	*p=0.0258
VIM	*p=0.0271
ITGAV	*p=0.0289
KRT19	*p=0.0337
WNT5B	*p=0.0454

Figure 2e

Comparison	Exact p value
siControl vs. si*SERPINE1*	****p<0.0001
siControl vs. si*WNT11*	****p<0.0001
siControl vs. si*WNT5B*	****p<0.0001
siControl vs. si*AHNAK*	****p<0.0001
siControl vs. si*TCF4*	****p<0.0001
siControl vs. si*CAV2*	****p<0.0001

Figure 2f

Comparison	Exact p value
siControl vs. si*SERPINE1*	****p<0.0001
siControl vs. si*WNT11*	****p<0.0001
siControl vs. si*WNT5B*	*p=0.0012
siControl vs. si*AHNAK*	****p<0.0001
siControl vs. si*TCF4*	****p<0.0001

Figure 2g

Comparison	Exact p value
siControl vs. si*SERPINE1*	***p=0.0007
siControl vs. si*WNT11*	*p=0.0387
siControl vs. si*WNT5B*	**p=0.0076
siControl vs. si*CAV2*	***p=0.0007

Figure 2h

Comparison	Exact p value
siControl vs. si*SERPINE1*	***p=0.0001
siControl vs. si*WNT11*	***p=0.0003
siControl vs. si*WNT5B*	***p=0.0003
siControl vs. si*AHNAK*	***p=0.0003

Figure 3a

Comparison	Exact p value
ALCAM	*p=0.0140
CD44	*p=0.0249
ALDH1A3	***p=0.0003
ALDH1A1	**p=0.0075
OCT4	*p=0.0437
JARID1B	p=0.0712
SOX2	p=0.143

Figure 3b

Comparison	Exact p value
Control vs. H1152	*p=0.0159
Control vs. GSK269962A	***p=0.0002
Control vs. Blebbistatin	***p=0.0004

Figure 3c

Comparison	Exact p value
Control vs. H1152	***p=0.0008
Control vs. GSK269962A	**p=0.0038
Control vs. Blebbistatin	**p=0.0035

Figure 3d

Comparison	Exact p value
A375M2 siControl vs. si*ROCK1/2*	**p=0.0074
A375M2 siControl vs. si*MYL9*	**p=0.0016
A375M2 siControl vs. si*MYL12B*	***p=0.0008
WM1361 siControl vs. si*ROCK1/2*	***p=0.0005
WM1361 siControl vs. si*MYL9*	****p<0.0001
WM1361 siControl vs. si*MYL12B*	****p<0.0001

Figure 3e

Comparison	Exact p value
A375M2 siControl vs. si*ROCK1/2*	**p=0.007
A375M2 siControl vs. si*MYL9*	**p=0.0031
A375M2 siControl vs. si*MYL12B*	**p=0.0036
WM1361 siControl vs. si*ROCK1/2*	*p=0.0424
WM1361 siControl vs. si*MYL9*	**p=0.0078
WM1361 siControl vs. si*MYL12B*	***p=0.0003

Figure 3g

Comparison	Exact p value
A375M2 siControl vs. si*WNT11*#1	*p=0.0131
A375M2 siControl vs. si*WNT11*#2	**p=0.0056
A375M2 siControl vs. si*WNT11*#3	**p=0.0068
WM1361 siControl vs. si*WNT11*#1	***p=0.0002
WM1361 siControl vs. si*WNT11*#2	***p=0.0002
WM1361 siControl vs. si*WNT11*#3	****p<0.0001

Figure 3h

Comparison	Exact p value
A375M2 siControl vs. si*WNT5B*	***p=0.0003
WM1361 siControl vs. si*WNT5B*	**p=0.0012

Figure 4a

Comparison	Exact p value	Exact p value	Exact p value	Exact p value
A375M2 vs	A375P	H1152	Y27632	Blebbistatin
FZD7	*p=0.0320	*p=0.0190	*p=0.0314	ns
RYK	ns	**p=0.0013	***p=0.0004	*p=0.0395
FZD3	ns	ns	ns	ns
FZD6	***p=0.0006	***p=0.0010	**p=0.0030	**p=0.0041
FZD1	***p=0.0008	*p=0.0102	**p=0.0070	**p=0.0078
ROR2	ns	ns	ns	*p=0.0148
FZD2	ns	ns	*p=0.0145	**p=0.0019
FZD9	ns	ns	ns	ns

Figure 4b

Comparison	Exact p value	Exact p value	Exact p value	Exact p value
Metastasis vs Primary	Xu	Riker	Kabbarah	TCGA
FZD7	*p=0.0141	*p=0.0232	*p=0.0251	ns
RYK	****p<0.0001	ns	*p=0.0172	*p=0.0131
FZD3	***p=0.0001	ns	****p<0.0001	ns
FZD6	**p=0.0011	ns	ns	ns
FZD1	ns	ns	*p=0.0262	***p=0.0004
ROR2	ns	ns	***p=0.0007	ns
FZD2	ns	ns	ns	ns
FZD9	ns	ns	ns	*p=0.0124

Figure 4c

Comparison	Exact p value
A375M2 siControl vs. si*FZD7*	**p=0.0022
WM1361 siControl vs. si*FZD7*	***p=0.0004
WM1361 siControl vs. si*RYK*	**p=0.0054

Figure 4e

Comparison	Exact p value
A375M2 siControl vs. si*FZD7*	****p<0.0001
A375M2 siControl vs. si*RYK*	****p<0.0001
WM1361 siControl vs. si*FZD7*	****p<0.0001
WM1361 siControl vs. si*RYK*	***p=0.0001

Figure 4f

Comparison	Exact p value
A375M2 siControl vs. si*FZD7*	*p=0.0441
A375M2 siControl vs. si*RYK*	*p=0.0403
WM1361 siControl vs. si*FZD7*	***p=0.0001
WM1361 siControl vs. si*RYK*	****p<0.0001

Figure 4g

Comparison	Exact p value
A375M2 siControl vs. si*FZD7*	***p=0.0006
WM1361 siControl vs. si*FZD7*	*p=0.0259

Figure 4h

Comparison	Exact p value
A375M2 siControl vs. si*PLCB1*	****p<0.0001
A375M2 siControl vs. si*DAAM1*	****p<0.0001
WM1361 siControl vs. si*PLCB1*	***p=0.0002
WM1361 siControl vs. si*DAAM1*	****p<0.0001

Figure 4i

Comparison	Exact p value
A375M2 siControl vs. si*DAAM1*	**p=0.0047
WM1361 siControl vs. si*PLCB1*	**p=0.0093
WM1361 siControl vs. si*DAAM1*	****p<0.0001

Figure 4k

Comparison	Exact p value
A375M2 siControl vs. si*DAAM1*	**p=0.0015
WM1361 siControl vs. si*DAAM1*	***p=0.0004

Figure 4l

Comparison	Exact p value
siControl vs. siControl+WNT11	****p<0.0001
si*PLCB1* vs. si*PLCB1*+WNT11	****p<0.0001
siControl vs. si*PLCB1*	**p=0.005
siControl vs. si*DAAM1*	**p=0.0012

Figure 4m

Comparison	Exact p value
siControl vs. siControl+WNT11	*p=0.0462

Figure 4n

Comparison	Exact p value
siControl vs. siControl+WNT11	*p=0.0112

Figure 5a

Comparison	Exact p value
Control vs. GSK269962A	**p=0.0092
Control vs. H1152	**p=0.0019

Figure 5b

Comparison	Exact p value
Control vs. GSK269962A	*p=0.0141
Control vs. H1152	**p=0.0063

Figure 5c

Comparison	Exact p value
Control vs. GSK269962A	**p=0.0019
Control vs. H1152	**p=0.0013

Figure 5d

Comparison	Exact p value
Control vs. GSK269962A	**p=0.0055
Control vs. H1152	***p=0.0004

Figure 5f

Comparison	Exact p value
shControl TB vs. shControl IF	***p=0.0003
shControl TB vs. sh*FZD7* TB	****p<0.0001
shControl IF vs. sh*FZD7* IF	****p<0.0001
sh*FZD7* TB vs. sh*FZD7* IF	*p=0.0196

Figure 5g

Comparison	Exact p value
shControl TB vs. shControl IF	****p<0.0001
shControl TB vs. sh*FZD7* TB	****p<0.0001
shControl IF vs. sh*FZD7* IF	****p<0.0001

Figure 5h

Comparison	Exact p value
shControl TB vs. shControl IF	***$p=0.0001$
shControl TB vs. sh*FZD7* TB	****$p<0.0001$
shControl IF vs. sh*FZD7* IF	****$p<0.0001$

Figure 5j

Comparison	Exact p value
shControl TB vs. shControl IF	****$p<0.0001$
shControl TB vs. sh*DAAM1* TB	****$p<0.0001$
shControl IF vs. sh*DAAM1* IF	****$p<0.0001$
sh*DAAM1* TB vs. sh*DAAM1* IF	*$p=0.0289$

Figure 5k

Comparison	Exact p value
shControl TB vs. shControl IF	****$p<0.0001$
shControl TB vs. sh*DAAM1* TB	**$p=0.0011$
shControl IF vs. sh*DAAM1* IF	****$p<0.0001$

Figure 5l

Comparison	Exact p value
shControl TB vs. shControl IF	**$p=0.0069$
shControl TB vs. sh*DAAM1* TB	**$p=0.0058$
shControl IF vs. sh*DAAM1* IF	***$p=0.0009$

Figure 5m

Comparison	Exact p value
24 h shControl vs. sh*DAAM1*	***$p=0.0001$
3 weeks shControl vs. sh*DAAM1*	*$p=0.0177$

Figure 6d

Comparison	Exact p value
Control TB vs. Control IF	***$p=0.0002$
Control TB vs. Control DIF	****$p<0.0001$
Control IF vs. Control DIF	****$p<0.0001$
Control IF vs. ROCKi IF	**$p=0.0064$
Control DIF vs. ROCKi DIF	**$p=0.0095$
ROCKi TB vs. ROCKi IF	*$p=0.0198$
ROCKi TB vs. ROCKi DIF	****$p<0.0001$
ROCKi IF vs. ROCKi DIF	****$p<0.0001$

Figure 6e

Comparison	Exact p value
Control TB vs. Control IF	****$p<0.0001$
Control TB vs. Control DIF	****$p<0.0001$
Control IF vs. Control DIF	**$p=0.0038$
Control IF vs. ROCKi IF	**$p=0.002$
Control DIF vs. ROCKi DIF	**$p=0.0036$
ROCKi TB vs. ROCKi IF	**$p=0.0027$
ROCKi TB vs. ROCKi DIF	****$p<0.0001$
ROCKi IF vs. ROCKi DIF	**$p=0.0036$

Figure 6e

Comparison	Exact p value
Control TB vs. Control IF	*$p=0.0139$
Control TB vs. Control DIF	****$p<0.0001$
Control IF vs. Control DIF	***$p=0.0002$
Control DIF vs. ROCKi DIF	**$p=0.0029$
ROCKi TB vs. ROCKi DIF	**$p=0.0019$
ROCKi IF vs. ROCKi DIF	*$p=0.0139$

Figure 6f

Comparison	Exact p value
Control TB vs. Control IF	*$p=0.0243$
Control TB vs. Control DIF	****$p<0.0001$
Control IF vs. Control DIF	**$p=0.0016$
Control TB vs. ROCKi TB	***$p=0.0007$
Control IF vs. ROCKi IF	**$p=0.0016$
Control DIF vs. ROCKi DIF	*$p=0.0452$
ROCKi TB vs. ROCKi IF	*$p=0.0167$
ROCKi TB vs. ROCKi DIF	****$p<0.0001$
ROCKi IF vs. ROCKi DIF	***$p=0.0001$

Figure 6j Comparison	Exact p value
Control vs. ROCKi	*$p=0.0107$

Figure 6k Comparison	Exact p value
Control vs. ROCKi	*$p=0.0317$

Figure 6l Comparison	Exact p value
Control vs. ROCKi	**$p=0.0022$

Figure 6m Comparison	Exact p value
Control vs. ROCKi	*$p=0.0411$

Supplementary Figure 1a

Comparison	Exact p value
A375M2 TB vs. A375M2 IF	****$p<0.0001$
A375M2 IF vs. A375P IF	***$p=0.0001$

Supplementary Figure 1c

Comparison	Exact p value
A375M2 TB vs. A375M2 IF	****$p<0.0001$
A375M2 TB vs. A375P TB	****$p<0.0001$
A375M2 IF vs. A375P IF	****$p<0.0001$
A375P TB vs. A375P IF	****$p<0.0001$

Supplementary Figure 1e

Comparison	Exact p value
A375M2 P1 vs. P4	***$p=0.0005$
A375M2 P2 vs. P4	***$p=0.001$
A375M2 P3 vs. P4	**$p=0.01$
WM983B P1 vs. P4	*$p=0.0296$

Supplementary Figure 1f

Comparison	Exact p value
A375M2 P1 vs. P3	**$p=0.0087$
A375M2 P1 vs. P4	***$p=0.0002$
A375M2 P2 vs. P4	**$p=0.0026$
WM983B P1 vs. P3	**$p=0.0011$
WM983B P1 vs. P4	****$p<0.0001$
WM983B P2 vs. P3	*$p=0.0144$
WM983B P2 vs. P4	***$p=0.0006$

Supplementary Figure 1g

Comparison	Exact p value
A375M2 P1 vs. P3	***p=0.0002
A375M2 P1 vs. P4	****p<0.0001
A375M2 P2 vs. P3	*p=0.0245
A375M2 P2 vs. P4	***p=0.0008
WM983B P1 vs. P3	**p=0.0044
WM983B P1 vs. P4	***p=0.0002
WM983B P2 vs. P4	**p=0.0096

Supplementary Figure 1h

Comparison	Exact p value
A375M2 P0 vs. P3	***p=0.0001
A375M2 P1 vs. P3	*p=0.0274
WM983B P0 vs. P1	**p=0.0054
WM983B P0 vs. P2	*p=0.0377
WM983B P0 vs. P3	***p=0.0002

Supplementary Figure 1i

Comparison	Exact p value
A375M2 P0 vs. P1	****p<0.0001
A375M2 P0 vs. P2	**p=0.0013
A375M2 P0 vs. P3	****p<0.0001
WM983B P0 vs. P1	*p=0.0101
WM983B P0 vs. P2	**p=0.0016
WM983B P0 vs. P3	****p<0.0001
WM983B P1 vs. P3	****p<0.0001
WM983B P2 vs. P3	****p<0.0001

Supplementary Figure 1j

Comparison	Exact p value
A375M2 P0 vs. P3	**p=0.0096
A375M2 P1 vs. P3	**p=0.0022
WM983B P0 vs. P3	***p=0.0003
WM983B P1 vs. P3	***p=0.0002

Supplementary Figure 1m

Comparison	Exact p value
GFP vs. MLC-GFP	**p=0.0016

Supplementary Figure 1n

Comparison	Exact p value
GFP vs. MLC-GFP	***p=0.0007

Supplementary Figure 1o

Comparison	Exact p value
GFP vs. MLC-GFP	**p=0.0044

Supplementary Figure 2d

Comparison	Exact p value
A375M2 siControl vs. si*WNT11*#1	***p=0.0006
A375M2 siControl vs. si*WNT11*#2	***p=0.0006
A375M2 siControl vs. si*WNT11*#3	**p=0.0055
WM1361 siControl vs. si*WNT11*#1	**p=0.0043
WM1361 siControl vs. si*WNT11*#2	**p=0.0092
WM1361 siControl vs. si*WNT11*#3	**p=0.0066

Supplementary Figure 2f

Comparison	Exact p value
A375M2 siControl vs. si*WNT11*#1	*p=0.0391
A375M2 siControl vs. si*WNT11*#2	*p=0.0115
A375M2 siControl vs. si*WNT11*#3	*p=0.0109
WM1361 siControl vs. si*WNT11*#1	*p=0.0113
WM1361 siControl vs. si*WNT11*#2	**p=0.0014
WM1361 siControl vs. si*WNT11*#3	**p=0.0024

Supplementary Figure 2g

Comparison	Exact p value
A375M2 siControl vs. si*WNT11*#1	**p=0.0035
A375M2 siControl vs. si*WNT11*#2	*p=0.0153
A375M2 siControl vs. si*WNT11*#3	*p=0.0276
WM1361 siControl vs. si*WNT11*#1	*p=0.0458
WM1361 siControl vs. si*WNT11*#2	*p=0.0289
WM1361 siControl vs. si*WNT11*#3	*p=0.0498

Supplementary Figure 2h

Comparison	Exact p value
siControl vs. si*WNT5B*	**p=0.0035

Supplementary Figure 3a

Comparison	Exact p value
JARID1B	***p=0.0005
ALDH1A3	**p=0.0025
SOX2	p=0.0559
CD44	**p=0.0046
OCT4	p=0.1202
ALCAM	p=0.557
ALDH1A1	p=0.616

Supplementary Figure 3d

Comparison	Exact p value
WM1361 Control vs. GSK	**p=0.0013
WM983B Control vs. GSK	**p=0.0034

Supplementary Figure 3e

Comparison	Exact p value
WM1361 Control vs. GSK	****p<0.0001
WM983B Control vs. GSK	*p=0.0303

Supplementary Figure 4c

Comparison	Exact p value
A375M2 shControl vs. sh*FZD7*#1	****p<0.0001
A375M2 shControl vs. sh*FZD7*#2	****p<0.0001
WM1361 shControl vs. sh*FZD7*#1	**p=0.0011
WM1361 shControl vs. sh*FZD7*#2	**p=0.0073

Supplementary Figure 4d

Comparison	Exact p value
A375M2 shControl vs. sh*FZD7*#1	**p=0.0033
A375M2 shControl vs. sh*FZD7*#2	**p=0.0046
WM1361 shControl vs. sh*FZD7*#1	**p=0.0067
WM1361 shControl vs. sh*FZD7*#2	**p=0.0061

Supplementary Figure 4h

**Comparison**	**Exact p value**	**Exact p value**	**Exact p value**	**Exact p value**
Metastasis vs Primary	Xu	Riker	Kabbarah	TCGA
PLCB1	***p=0.0002	ns	***p=0.0002	*p=0.0312
PLCB2	ns	ns	*p=0.0209	****p<0.0001
PLCB3	ns	*p=0.0323	ns	ns
PLCB4	ns	ns	*p=0.0115	****p<0.0001

Supplementary Figure 4i

**Comparison**	**Exact p value**
A375M2 siControl vs. si*PLCB1*	****p<0.0001
A375M2 siControl vs. si*DAAM1*	****p<0.0001
WM1361 siControl vs. si*PLCB1*	****p<0.0001
WM1361 siControl vs. si*DAAM1*	***p=0.001

Supplementary Figure 4l

**Comparison**	**Exact p value**
A375M2 shControl vs. sh*DAAM1*#1	**p=0.0047
A375M2 shControl vs. sh*DAAM1*#2	*p=0.0308
WM1361 shControl vs. sh*DAAM1*#1	****p<0.0001
WM1361 shControl vs. sh*DAAM1*#2	***p=0.0004

Supplementary Figure 4n

**Comparison**	**Exact p value**
A375M2 shControl vs. sh*DAAM1*#1	***p=0.0002
A375M2 shControl vs. sh*DAAM1*#2	****p<0.0001
WM1361 shControl vs. sh*DAAM1*#1	****p<0.0001
WM1361 shControl vs. sh*DAAM1*#2	****p<0.0001

Supplementary Figure 4p

**Comparison**	**Exact p value**
NANOG	**p=0.0026
OCT4	*p=0.0105
SOX2	**p=0.0046
CD44	*p=0.0205
ALCAM	**p=0.0011
JARID1B	p=0.0716
ALDH1A1	p=0.1855
ALDH1A3	p=0.3215

Supplementary Figure 5d

**Comparison**	**Exact p value**
Control vs. GSK269962A 5µM	***p=0.0003
Control vs. GSK269962A 1µM	*p=0.0343

Supplementary Figure 5e

**Comparison**	**Exact p value**
Control vs. GSK269962A	**p=0.0044

Supplementary Figure 5f

**Comparison**	**Exact p value**
Control vs. GSK269962A	***p=0.0008

Supplementary Figure 5g

**Comparison**	**Exact p value**
Control vs. GSK269962A	**p=0.0076

Supplementary Figure 5j

**Comparison**	**Exact p value**
shControl vs. sh*DAAM1*	***p=0.0007

Supplementary Figure 5m

**Comparison**	**Exact p value**
shControl TB vs. shControl IF	****p<0.0001
shControl TB vs. sh*DAAM1* TB	*p=0.0124
shControl IF vs. sh*DAAM1* IF	*p=0.0228
sh*DAAM1* TB vs. sh*DAAM1* IF	****p<0.0001

Supplementary Figure 5n left

**Comparison**	**Exact p value**
shControl TB vs. shControl IF	****p<0.0001
shControl TB vs. sh*DAAM1* TB	*p=0.0137
shControl IF vs. sh*DAAM1* IF	***p=0.0003
sh*DAAM1* TB vs. sh*DAAM1* IF	***p=0.0004

Supplementary Figure 5n right

**Comparison**	**Exact p value**
shControl TB vs. shControl IF	****p<0.0001
shControl IF vs. sh*DAAM1* IF	***p=0.0003

Supplementary Figure 5o

**Comparison**	**Exact p value**
shControl TB vs. shControl IF	****p<0.0001
shControl TB vs. sh*DAAM1* TB	***p=0.0008
shControl IF vs. sh*DAAM1* IF	****p<0.0001
sh*DAAM1* TB vs. sh*DAAM1* IF	***p=0.0009

Supplementary Figure 7f

**Comparison**	**Exact p value**
A375M2 siControl vs. si*ALDH1A1*	****p<0.0001
WM1361 siControl vs. si*ALDH1A1*	*p=0.0230

Supplementary Figure 7g

**Comparison**	**Exact p value**
A375M2 siControl vs. si*ALDH1A1*	****p<0.0001
WM1361 siControl vs. si*ALDH1A1*	**p=0.0082

Supplementary Figure 7j

**Comparison**	**Exact p value**
A375M2 siControl vs. si*ALDH1A1*	*p=0.0248
WM1361 siControl vs. si*ALDH1A1*	*p=0.0411

Supplementary Table 2. Clinical information for primary melanoma patients

	Patients (n = 53)
**Age, years (mean ± SD)**	65 ± 17.5
**Gender**	
Male	30 (56.6%)
Female	23 (43.4%)
**Stage**	
II	13 (24.5%)
III	31 (58.5%)
IV	6 (11.3%)
n.a.	3 (5.7%)
**Location**	
Trunk	23 (43.4%)
Head and Neck	15 (28.3%)
Lower Limb	8 (15.1%)
Upper Limb	5 (9.4%)
Foot	2 (3.8%)

Supplementary Table 3. Clinical information for metastatic melanoma patients

	Patients (n = 45)
**Age, years (mean ± SD)**	63 ± 16.3
**Gender**	
Male	26 (57.8%)
Female	19 (42.2%)
**Location**	
Lymph Node	39 (86.7%)
Cutaneous/ subcutaneous	5 (11.1%)
Lung	1 (2.2%)

Supplementary Table 4. List of siRNA sequences.

Target	siRNA sequence
Control siRNA (siGenome Non-Targeting siRNA #5 or OT non-targeting siRNA #1)	5’-UGGUUUACAUGUCGACUAA-3’
SERPINE1	siGENOME SMARTpool:<br>5’-GCUAUGGGAUUCAAGAUUG-3’<br>5’-GGAGCACGGUCAAGCAAGU-3’<br>5’-CUAGAGAACCUGGGAAUGA-3’<br>5’-CGACAUGUUCAGACAGUUU-3’
WNT11	#1: siGENOME SMARTpool:<br>5’-CAGGAUCCCAAGCCAAUAA-3’<br>5’-CGACAGCUGCGACCUUAUG-3’<br>5’-GUCGAGCGGUGCCACUGUA-3’<br>5’-GGACUCGGAACUCGUCUAU-3’<br>ON-TARGETplus individual sequences:<br>#2 OT6: 5’-GCGCUAUGUCUGCAAGUGA-3’<br>#3 OT7: 5’-AACAAGACAUCCAACGGAA-3’
WNT5B	siGENOME SMARTpool:<br>5’-GCAGGGCUGUGUAUAAGAU-3’<br>5’-AGAGGAAGCUGUGCCAAUU-3’<br>5’-GACCCGAGAUGUUUAUCAU-3’<br>5’-AGACGUAGCCUGCAAAUGC-3’
AHNAK	siGENOME SMARTpool:<br>5’-GCCUGAAGCUGCACCGCAA-3’<br>5’-GGGACCAGAUUGUGGGUGC-3’<br>5’-GCGUCUUUGUGCAGGAGGU-3’<br>5’-UGACCAUCGCCCAGAGGGA-3’
TCF4	siGENOME SMARTpool:<br>5’-GAAAUUAGAUGACGACAAG-3’<br>5’-ACAAAGAGCUGAGUGAUUU-3’<br>5’-GGGCAACUCUUCUCAUAUU-3’<br>5’-GCACUUGCUUCGAUCUAUU-3’
CAV2	siGENOME SMARTpool:<br>5’-GUAAAGACCUGCCUAAUGG-3’<br>5’-UAUCAUUGCUCCAUUGUGU-3’<br>5’-GGACGUACAGCUCUUCAUG-3’<br>5’-CGGCUCAACUCGCAUCUCA-3’
ROCK1	ON-TARGETplus individual sequence:<br>OT8: 5’-CCAGGAAGGUAUAUGCUAU-3’
ROCK2	ON-TARGETplus individual sequence:<br>OT8: 5’-GAAACUAAUAGGACACUAA-3’
MYL9	ON-TARGETplus individual sequence:<br>OT5: 5’-CCAAGGAUAAAGACGACUA-3’
MYL12B	ON-TARGETplus SMARTpool:<br>5’-CCACUUAGCACUUGUAUAA-3’<br>5’-GGGUGUAAAUUGUAUUGAA-3’<br>5’-CCUCAUAGAACCUGUUGCA-3’<br>5’-UGUAUUUAUUCCAGACCUU-3’
FZD7	siGENOME SMARTpool:<br>5’-UCAAGUACCUGAUGACCAU-3’<br>5’-GUUCGUCUACCUCUUCAUA-3’<br>5’-CAUAGGCACGUCCUUCUUG-3’<br>5’-UGAUGUACUUUAAGGAGGA-3’
RYK	siGENOME SMARTpool:<br>5’-GAAAGAUGGUUACCGAAUA-3’<br>5’-GGUGAAGGAUAUAGCAAUA-3’<br>5’-CGAAGUCCAAGGUUGAAUA-3’<br>5’-AGUAAUAUUUCUCGUAGCA-3’
PLCB1	siGENOME SMARTpool:<br>5’-CAACAGAAAUCGUUUGUGA-3’<br>5’-GAUGAUGACUCAACUAUUG-3’<br>5’-GCAAUUGGCUGCUUUGACA-3’<br>5’-GAAGAUAACAGAAGCUAAA-3’
DAAM1	siGENOME SMARTpool:<br>5’-GAGAUAAGUUUGUGUCUGU-3’<br>5’-GUACGAAUGUUGGUUAAUG-3’<br>5’-CGGAAUCGCAAACGUAUUA-3’<br>5’-GAGCCGAAUUAAUCACUAU-3’
ALDH1A1	siGENOME SMARTpool:<br>5’-GGACAAUGCUGUUGAAUUU-3’<br>5’-GCACUGAGCUGUGGAAACA-3’<br>5’-CCACGUGGCAUCUUUAAUA-3’<br>5’-GAACAGUGUGGGUGAAUUG-3’

Supplementary Table 5. Detailed information about immunohistochemical stainings

Antibody	Antibody #Catalog	Clone	Primary antibody conditions	Antigen Retrieval	Detection Method #Catalog
**p-MLC2**	Cell Signaling #3671	Polyclonal	Rabbit, 1:50, 40 min, Room Temperature (RT)	10 min at 100°C, pH 6 Citrate Buffer H-3300	VECTASTAIN® ABC HRP Kit #PK-4000
**mCherry**	Abcam #ab167453	Polyclonal	Rabbit, 1:500, 20 min, RT	10 min at 100°C, pH 9 Tris Buffer H-3301	VECTASTAIN® ABC HRP Kit #PK-4000
**DAAM1**	Abcam #ab71327	Polyclonal	Rabbit, 1:300, 40 min, RT	10 min at 100°C, pH 6 Citrate Buffer H-3300	VECTASTAIN® ABC HRP Kit #PK-4000
**WNT11**	Novus Biologicals #NBP1-31406	Polyclonal	Rabbit, 1:200, 20 min, RT	10 min at 100°C, pH 9 Tris Buffer H-3301	EnVision Detection Systems Peroxidase/DAB, Rabbit/Mouse, HRP. Rabbit/Mouse (DAB+) #K500711
**WNT5B**	Abcam #ab115563	Polyclonal	Rabbit, 1:200, 20 min, RT	10 min at 100°C, pH 9 Tris Buffer H-3301	EnVision Detection Systems Peroxidase/DAB, Rabbit/Mouse, HRP. Rabbit/Mouse (DAB+) #K500711
**ALDH1A1**	BD Biosciences #611194	44/ALDH	Mouse, 1:100, 20 min, RT	10 min at 100°C, pH 6 Citrate Buffer H-3300	EnVision Detection Systems Peroxidase/DAB, Rabbit/Mouse, HRP. Rabbit/Mouse (DAB+) #K500711
**CD44**	Thermo Fisher #MA1-10225	IM7	Rat, 1:400, 40 min, RT	10 min at 100°C, pH 6 Citrate Buffer H-3300	VECTASTAIN® ABC HRP Kit #PK-4000
**NANOG**	Abcam #ab80892	Polyclonal	Rabbit, 1:100, 30 min, RT	10 min at 100°C, pH 9 Tris Buffer H-3301	EnVision Detection Systems Peroxidase/DAB, Rabbit/Mouse, HRP. Rabbit/Mouse (DAB+) #K500711
**ALDH1A3**	Novus Biologicals #NBP2-15339	Polyclonal	Rabbit, 1:200, 20 min, RT	10 min at 100°C, pH 9 Tris Buffer H-3301	EnVision Detection Systems Peroxidase/DAB, Rabbit/Mouse, HRP. Rabbit/Mouse (DAB+) #K500711
**OCT4**	Abcam #ab181557	EPR17929	Rabbit, 1:100, 30 min, RT	10 min at 100°C, pH 9 Tris Buffer H-3301	EnVision Detection Systems Peroxidase/DAB, Rabbit/Mouse, HRP. Rabbit/Mouse (DAB+) #K500711
**ki-67 (Human)**	Dako_Agillent #GA626	MIB-1	Mouse, 1:500, 40 min, RT	10 min at 100°C, pH 6 Citrate Buffer H-3300	VECTASTAIN® ABC HRP Kit #PK-4000
**ki-67 (Mouse)**	Dako_Agillent #M7249	TEC-3	Rat, 1:200, 40 min, RT	10 min at 100°C, pH 6 Citrate Buffer H-3300	VECTASTAIN® ABC HRP Kit #PK-4000
**FZD7**	Abcam #ab64636	Polyclonal	Rabbit, 1:200, 40 min, RT	10 min at 100°C, pH 9 Tris Buffer H-3301	VECTASTAIN® ABC HRP Kit #PK-4000

SUPPLEMENTARY REFERENCES

1. Verfaillie A, *et al.* Decoding the regulatory landscape of melanoma reveals TEADS as regulators of the invasive cell state. *Nat Commun* **6**, 6683 (2015).
2. Sanz-Moreno V, *et al.* ROCK and JAK1 signaling cooperate to control actomyosin contractility in tumor cells and stroma. *Cancer Cell* **20**, 229-245 (2011).
3. Hoek KS, *et al.* Metastatic potential of melanomas defined by specific gene expression profiles with no BRAF signature. *Pigment Cell Res* **19**, 290-302 (2006).
