# Supplementary material for: WNT11-FZD7-DAAM1 signalling supports tumour initiating abilities and melanoma amoeboid invasion
Source: Nat Commun. 2020 Oct 20;11:5315. doi: 10.1038/s41467-020-18951-2 (PMC7575593; doi:10.1038/s41467-020-18951-2)
Supplement: Supplementary file 3 — Reporting Summary [file 41467_2020_18951_MOESM3_ESM.pdf]

natureresearch

Corresponding author(s): Victoria Sanz-Moreno

Last updated by author(s): Aug 15, 2020

Reporting Summary

Nature Research wishes to improve the reproducibility of the work that we publish. This form provides structure for consistency and transparency in reporting. For further information on Nature Research policies, see Authors & Referees and the Editorial Policy Checklist.

Statistics

For all statistical analyses, confirm that the following items are present in the figure legend, table legend, main text, or Methods section.

n/a	Confirmed	
☐	☒	The exact sample size (*n*) for each experimental group/condition, given as a discrete number and unit of measurement
☐	☒	A statement on whether measurements were taken from distinct samples or whether the same sample was measured repeatedly
☐	☒	The statistical test(s) used AND whether they are one- or two-sided<br>*Only common tests should be described solely by name; describe more complex techniques in the Methods section.*
☒	☐	A description of all covariates tested
☐	☒	A description of any assumptions or corrections, such as tests of normality and adjustment for multiple comparisons
☐	☒	A full description of the statistical parameters including central tendency (e.g. means) or other basic estimates (e.g. regression coefficient) AND variation (e.g. standard deviation) or associated estimates of uncertainty (e.g. confidence intervals)
☐	☒	For null hypothesis testing, the test statistic (e.g. *F*, *t*, *r*) with confidence intervals, effect sizes, degrees of freedom and *P* value noted<br>*Give P values as exact values whenever suitable.*
☒	☐	For Bayesian analysis, information on the choice of priors and Markov chain Monte Carlo settings
☒	☐	For hierarchical and complex designs, identification of the appropriate level for tests and full reporting of outcomes
☒	☐	Estimates of effect sizes (e.g. Cohen's *d*, Pearson's *r*), indicating how they were calculated

Our web collection on statistics for biologists contains articles on many of the points above.

Software and code

Policy information about availability of computer code

Data collection	Phase contrast images were acquired with Zeiss Axio Vert.A inverted microscope and confocal images with Zeiss LSM 510 Meta confocal and Zeiss LSM 710 confocal microscopes and Zen 2.1 SP1 (black) software (Carl Zeiss). BD LSRFortessa™ system (BD Biosciences) and FlowJo 10.6 software were used to collect flow cytometry data. RT-PCR data was obtained using QuantStudio 7 Flex Real-Time PCR System and QuantStudio 1.3 Software (Applied Biosystems). Whole tissue sections were scanned and images obtained using NanoZoomer S210 slide scanner (Hamamatsu) and QuPath 0.1.2 software. Representative TMA pictures according to mean H-score were imaged using iScope microscope (IS.1159EPLi, Euromex) and ImageFocus 4.0 (Euromex) software.
Data analysis	Gene expression datasets were analysed using GSEA software (http://www.broadinstitute.org/gsea/index.jsp), ssGSEA Projection Software from GenePattern platform (https://www.broadinstitute.org/cancer/software/genepattern) and Ingenuity Pathway Analysis (IPA version 52912811) (Qiagen). Heatmaps were performed using MeV_4_9_0 software. Principal component analysis and survival analyses were performed using SPSS Statistics 25 (IBM) software. All other statistical analyses were performed using GraphPad Prism 8 (GraphPad Software, Inc). Limiting dilution assays were analysed using ELDA software (http://bioinf.wehi.edu.au/software/elda/). ImageJ 1.52e sofware was used to analyse microscopy images and quantify western blots. FlowJo 10.6 software was used to analyse flow cytometry data. IHC analyses (p-MLC2, ki-67, FZD7 and DAAM1 stainings) were performed using QuPath 0.1.2 software.

For manuscripts utilizing custom algorithms or software that are central to the research but not yet described in published literature, software must be made available to editors/reviewers. We strongly encourage code deposition in a community repository (e.g. GitHub). See the Nature Research guidelines for submitting code & software for further information.

Data

Policy information about availability of data

All manuscripts must include a data availability statement. This statement should provide the following information, where applicable:

- Accession codes, unique identifiers, or web links for publicly available datasets
- A list of figures that have associated raw data
- A description of any restrictions on data availability

Gene expression datasets re-analysed in this study are available from NCBI GEO under accession numbers: GSE23764, GSE8401, GSE7553 and GSE46517. Uncropped immunoblot scans for representative images displayed in the figures are shown in Source data. All other relevant data supporting the findings of this study are available within the article and its supplementary information and from the corresponding author on reasonable request.

Field-specific reporting

Please select the one below that is the best fit for your research. If you are not sure, read the appropriate sections before making your selection.

☒ Life sciences ☐ Behavioural & social sciences ☐ Ecological, evolutionary & environmental sciences

For a reference copy of the document with all sections, see nature.com/documents/nr-reporting-summary-flat.pdf

Life sciences study design

All studies must disclose on these points even when the disclosure is negative.

Sample size	No statistical methods were used to calculate sample size. Sample size was chosen based on standards in the field and previous experiments conducted in our laboratory (Sanz-Moreno et al, Cell 2008; Sanz-Moreno et al, Cancer Cell 2011; Orgaz et al, Nat Commun 2014; Cantelli et al, Curr Biol 2015; Herraiz et al, J Natl Cancer Inst 2015; Georgouli et al, Cell 2019; Orgaz et al, Cancer Cell 2020). The sample size was determined to be sufficient based on the size and consistency of the measurable differences between the groups. For in vitro experiments, at least three biologically independent experiments were performed. For animal experiments, a minimum of n=4-6 mice in each group/experiment were included.
Data exclusions	No data were excluded.
Replication	The data was reproduced in at least three independent biological experiments as specified in the figure legends.
Randomization	In vitro experiments were not randomized and this was not required for our study. Samples were allocated based on the treatments/experimental conditions applied and their identity was known during experimentation and data analysis. For in vivo experiments, mice of similar age were randomly allocated into experimental groups.
Blinding	The investigators were not blinded to allocation during experiments and outcome assessment. The results of the experiments provided quantitative data that was analysed with the appropriate statistical tests to evaluate differences and statistical significance, so blinding was not relevant. Immunohistochemical analyses were blinded to clinical patient information.

Reporting for specific materials, systems and methods

We require information from authors about some types of materials, experimental systems and methods used in many studies. Here, indicate whether each material, system or method listed is relevant to your study. If you are not sure if a list item applies to your research, read the appropriate section before selecting a response.

Materials & experimental systems

n/a	Involved in the study	
☐	☒	Antibodies
☐	☒	Eukaryotic cell lines
☒	☐	Palaeontology
☐	☒	Animals and other organisms
☐	☒	Human research participants
☒	☐	Clinical data

Methods

n/a	Involved in the study	
☒	☐	ChIP-seq
☐	☒	Flow cytometry
☒	☐	MRI-based neuroimaging

Antibodies

Antibodies used	Antibodies for Western blot included: pThr18/Ser19-MLC2 (1:750, #3674), MLC2 (1:750, #3672) and RhoA (1:1000, #2117) from Cell Signalling Technology; GFP (1:10000, sc-8334) from Santa Cruz Biotechnology; GAPDH (1:10000, MAB374) from Millipore. Antibodies and molecular probes for immunofluorescence included: pSer19-MLC2 (#3671, 1:200) from Cell Signalling; Alexa

	Fluor 546 Phalloidin (#A22283), Secondary Antibody Alexa Fluor 488 (#A-11034) and 647 (#A-21245) from Life Technologies. Detailed information about antibodies for IHC can be found in Supplementary Table 5.
Validation	All antibodies were commercially validated for the application used and have been previously used in relevant publications as for example: Ye et al, Nat Commun 2013; Herraiz et al, J Natl Cancer Inst 2015; Cheloufi et al, Nature 2015; Sarvi et al, Cell Chemical Biology 2018; Georgouli et al, Cell 2019; Dirice et al, Nat Metab 2019; Orgaz et al, Cancer Cell 2020.<br>p-MLC2, MLC2, ROCK1 and ROCK2 antibodies were tested for western blot analysis in cells transfected with siRNAs against MLC2, ROCK1 or ROCK2, respectively.<br>p-MLC2 antibody was validated for IHC analysis in paraffin-embedded cell pellets of cells transfected with siRNA against MLC2.<br>WNT11 and WNT5B antibodies were tested for IHC analysis in paraffin-embedded cell pellets of cells transfected with siRNAs against WNT11 or WNT5B, respectively.<br>DAAM1 and FZD7 antibodies were tested for IHC analysis in derived tissues from cells stably expressing shRNAs against DAAM1 or FZD7, respectively.<br>Stem-cell related antibodies were assessed for IHC analysis in seminoma, a germ cell derived tumour.

Eukaryotic cell lines

Policy information about cell lines

Cell line source(s)	A375P and A375M2 cells (Clark et al, Nature 2000) were from Prof Richard Hynes (HHMI, MIT, USA); WM1361 cells (Coriell WC00075) were from Prof Richard Marais (Cancer Research UK Manchester Institute, UK); WM983B (Coriell WC00066) and WM983A (Coriell WC00048) were purchased from Coriell Institute; B16F10 cells (ATCC CRL-6475) were from Dr Hector Peinado (CNIO, Spain); 4599 cells (Dhomen et al, Cancer Cell 2009; Hirata et al, Cancer Cell 2015) were from Dr Amine Sadok (Institute of Cancer Research, UK) and Prof Richard Marais; HEK293T cells (ATCC CRL-3216;) were from Dr Jeremy Carlton (The Francis Crick Institute, UK); HaCaT cells (DSMZ ACC-771) were from Dr Ester Martin-Villar (IIBM, UAM, Spain).
Authentication	A375M2, A375P, WM983B, WM983A and 4599 were authenticated using short tandem repeat DNA profiling. HEK293T, HaCaT, WM1361 and B16F10 were not authenticated. All cell lines were kept in culture for a maximum of three to four passages, and cell phenotypes were verified routinely.
Mycoplasma contamination	All cell lines tested negative for mycoplasma contamination.
Commonly misidentified lines<br>(See ICLAC register)	No commonly misidentified lines were used in this study.

Animals and other organisms

Policy information about studies involving animals; ARRIVE guidelines recommended for reporting animal research

Laboratory animals	Mouse model, NOD/SCID/IL2Rγ-/- (NSG), male and female, 6-12 week-old.<br>Mouse model, C57BL/6J, female, 8-12 week-old.
Wild animals	No wild animals were used in this study.
Field-collected samples	No field-collected samples were used in this study.
Ethics oversight	All animal experiments were approved by the Ethical Review Process Committees at Barts Cancer Institute, King's College London and The Francis Crick Institute and carried out under licences from the Home Office, UK.

Note that full information on the approval of the study protocol must also be provided in the manuscript.

Human research participants

Policy information about studies involving human research participants

Population characteristics	Detailed information of the patients included in this study can be found in Supplementary Tables 2 and 3.
Recruitment	Patients diagnosed of melanoma were recruited in Dermatology departments from Hospital Arnau de Vilanova (Lleida, Spain) and Hospital de Bellvitge (Barcelona, Spain) during the period 1992 to 2014. No selection on age or gender were made. All cases were diagnosed in the respective Pathology Departments as melanoma according to the latest AJCC criteria. Samples were collected with specific informed consent.
Ethics oversight	Tumour samples were processed by IRBLleida (PT17/0015/0027) and HUB-ICO-IDIBELL (PT17/0015/0024) Biobanks integrated in the Spanish National Biobank Network and Xarxa de Bancs de Tumors de Catalunya following standard operating procedures with the appropriate approval of the Ethics and Scientific Committee.

Note that full information on the approval of the study protocol must also be provided in the manuscript.

Flow Cytometry

Plots

Confirm that:

☒ The axis labels state the marker and fluorochrome used (e.g. CD4-FITC).

☒ The axis scales are clearly visible. Include numbers along axes only for bottom left plot of group (a 'group' is an analysis of identical markers).

☒ All plots are contour plots with outliers or pseudocolor plots.

☒ A numerical value for number of cells or percentage (with statistics) is provided.

Methodology

Sample preparation	Cells were harvested and incubated with 5 µM CellROX Green Reagent (Life Technologies) at 37ºC for 30 min in the dark. Then cells were diluted with FACS buffer (PBS-/-, 1% BSA, 2 mM EDTA, 0.1% NaN3) and fluorescence was analysed by flow cytometry.
Instrument	BD LSRFortessa™ system.
Software	FlowJo software.
Cell population abundance	10,000 cells were acquired and analyzed for each sample in each biological experiment.
Gating strategy	Cells were first gated on FSC-A vs SSC-A to eliminate cell debris. Then, discrimination of doublets was performed on FSC-A vs FSC-H and SSC-A vs SSC-W. Only viable and single cells were used for the analysis. CellROX green positivity was analysed using mean fluorescence intensity.

☒ Tick this box to confirm that a figure exemplifying the gating strategy is provided in the Supplementary Information.
