# Supplementary material for: WNT11-FZD7-DAAM1 signalling supports tumour initiating abilities and melanoma amoeboid invasion
Source: Nat Commun. 2020 Oct 20;11:5315. doi: 10.1038/s41467-020-18951-2 (PMC7575593; doi:10.1038/s41467-020-18951-2)

Rodriguez-Hernandez *et al*. Uncropped immunoblot images

Figure 2b

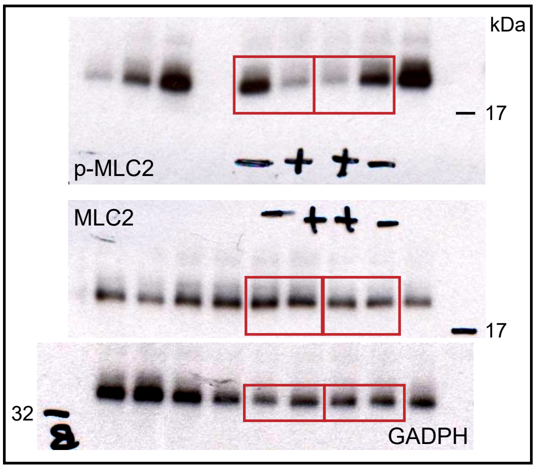

Figure 4m

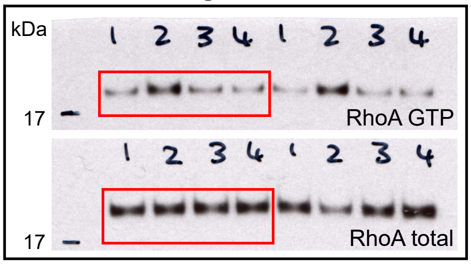

Figure 4n

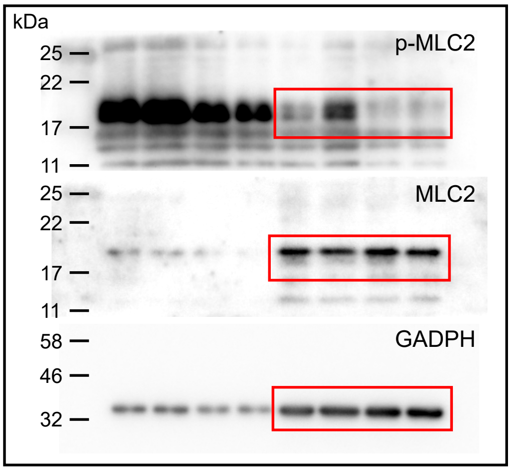

Supplementary Fig.1d

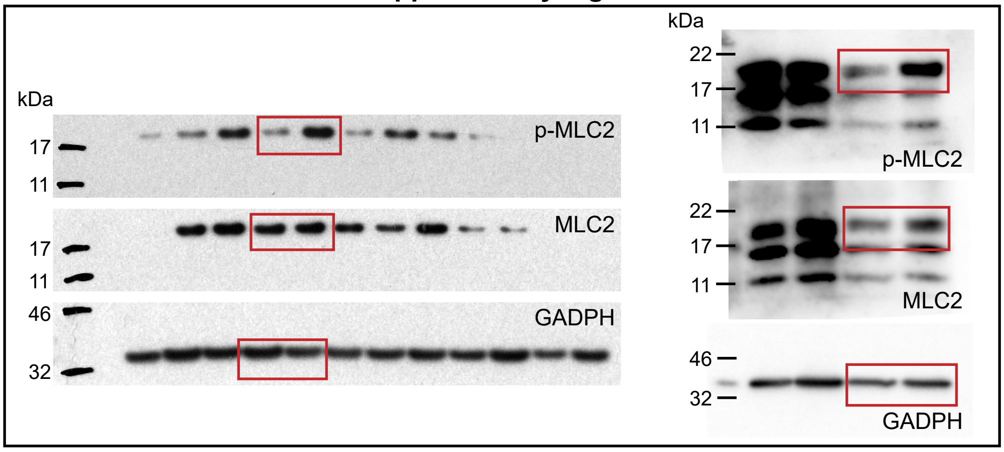

Supplementary Fig.1k

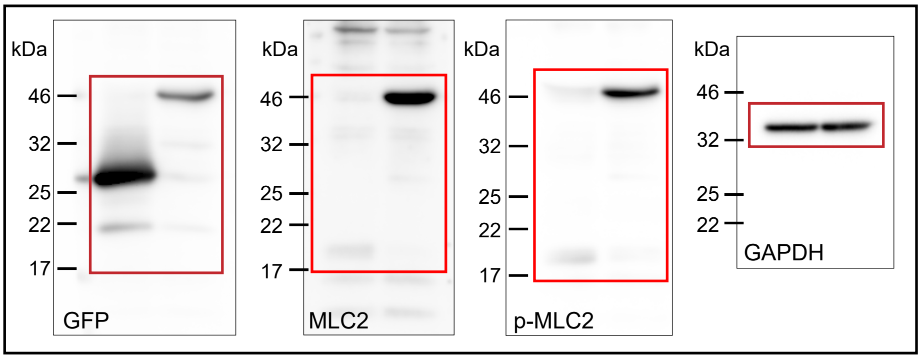

Rodriguez-Hernandez *et al.* Uncropped immunoblot images (continued)

Supplementary Fig.2f

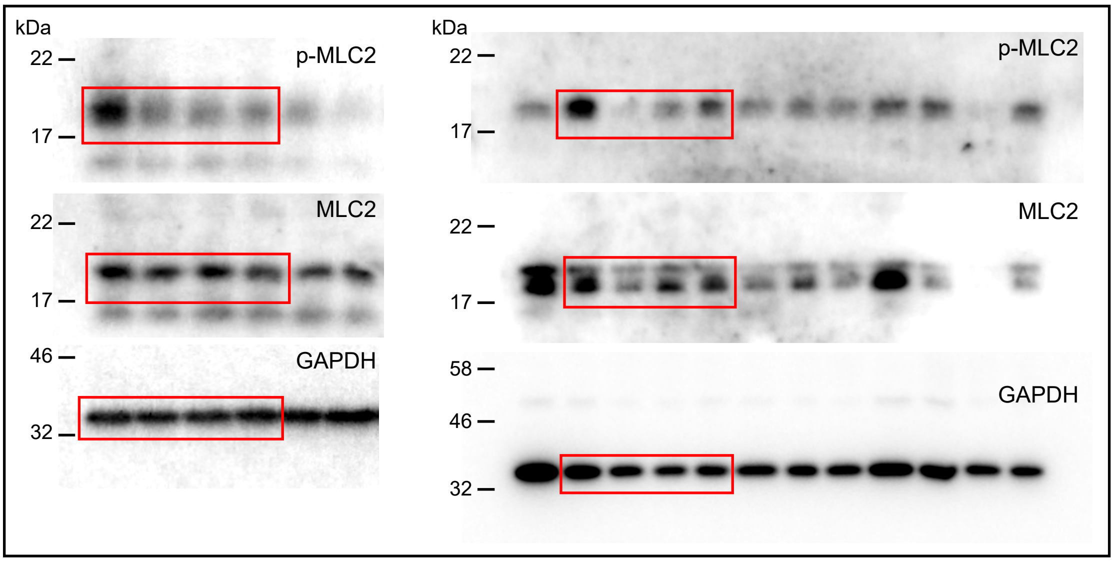

Supplementary Fig.2j

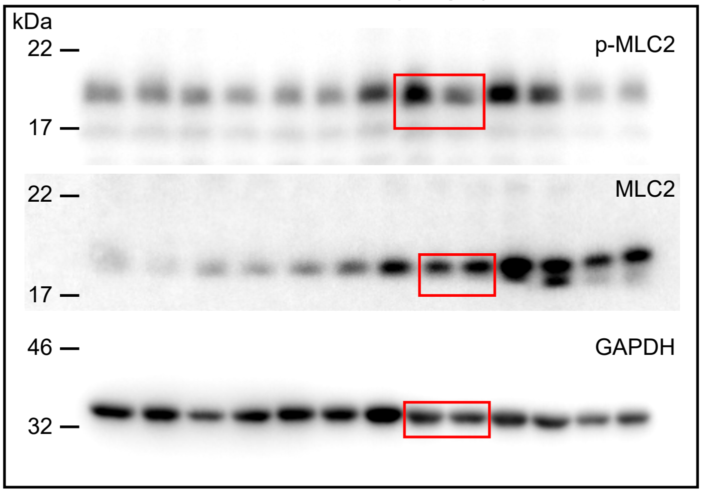

Supplementary Fig.3c

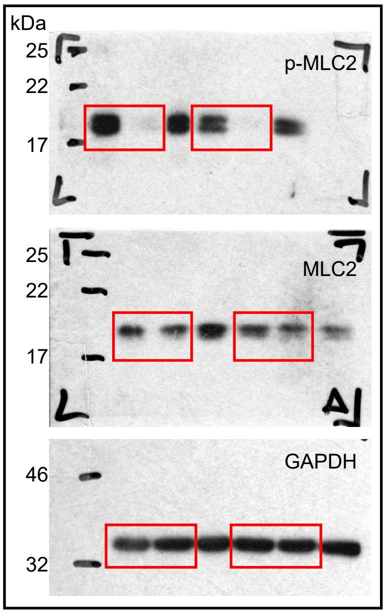

Supplementary Fig.4b

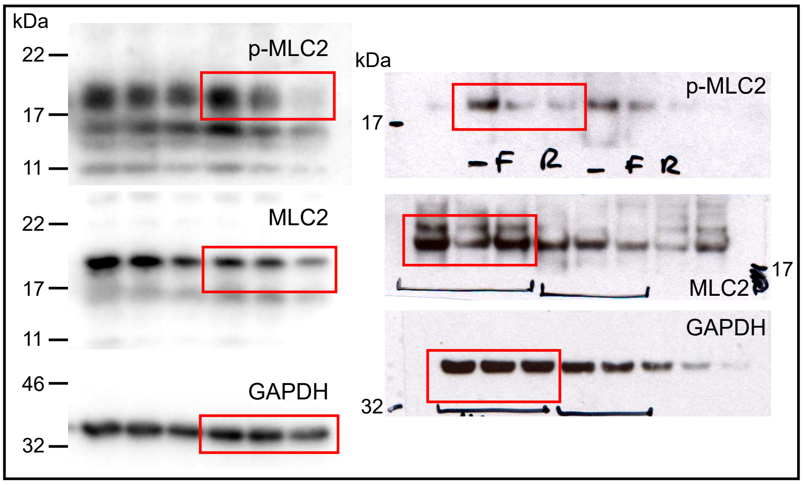

Rodriguez-Hernandez *et al*. Uncropped immunoblot images (continued)

Supplementary Fig.4g

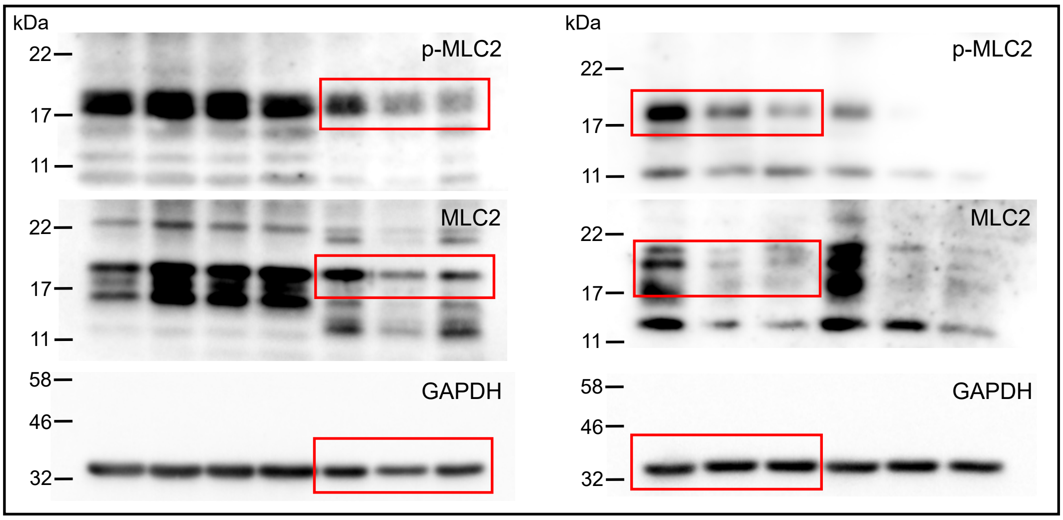

Supplementary Fig.4j

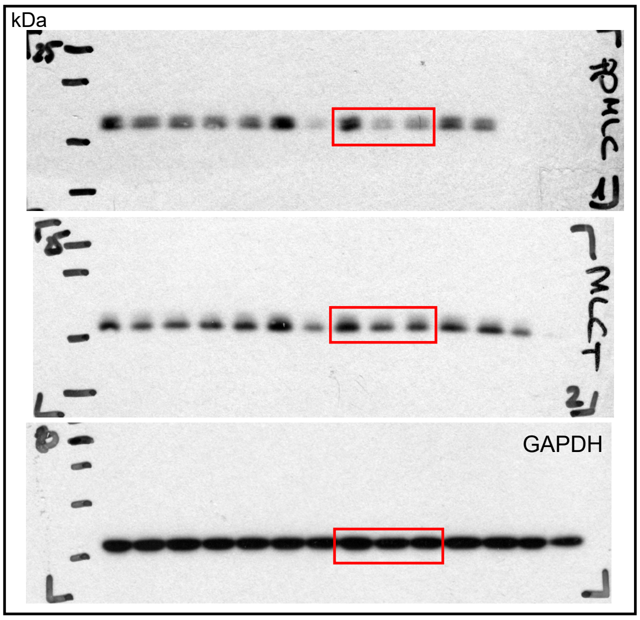

Supplementary Fig.4o

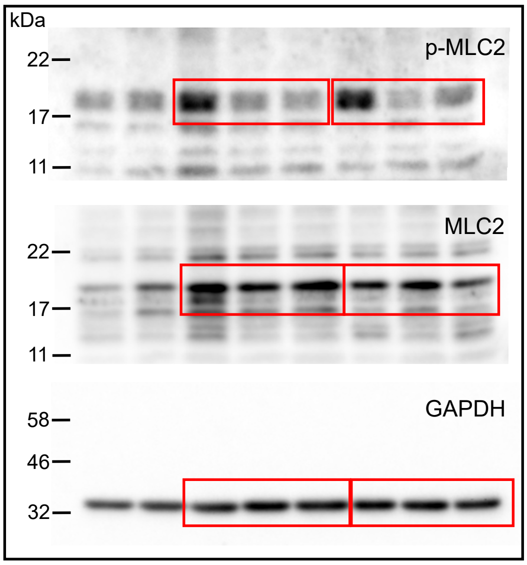

Supplementary Fig.7i

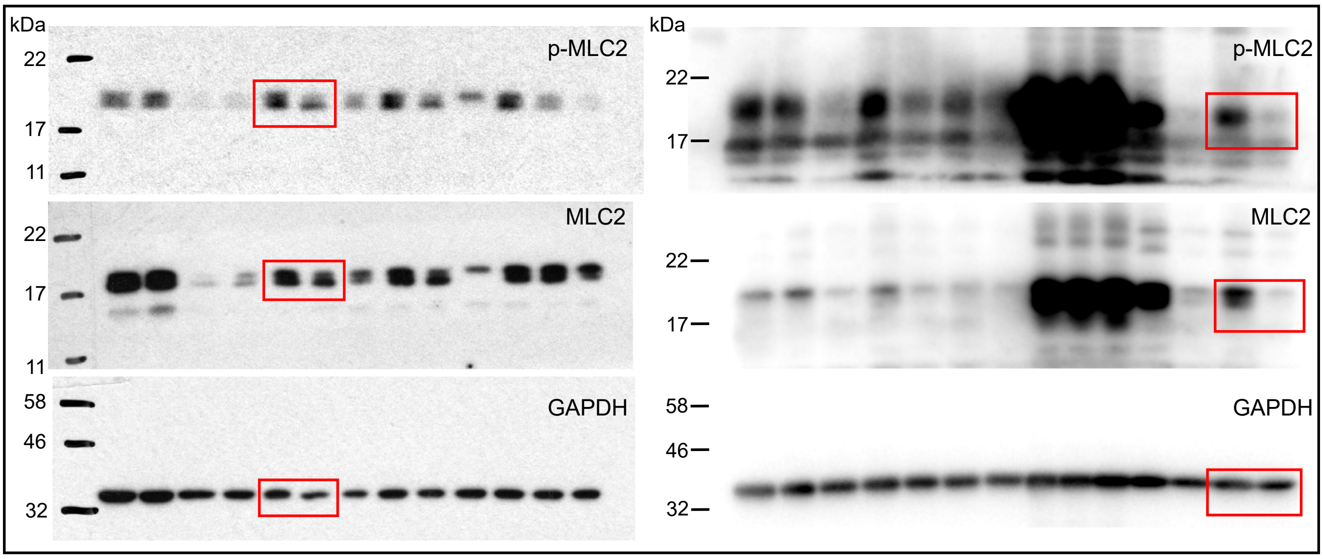

Supplement: Supplementary file 4 — Source Data [file 41467_2020_18951_MOESM4_ESM.pdf]
